# Inhibition of Platelet-Derived Growth Factor Receptor Signaling Regulates Oct4 and Nanog Expression, Cell Shape, and Mesenchymal Stem Cell Potency

**DOI:** 10.1002/stem.1015

**Published:** 2012-02-14

**Authors:** Stephen G Ball, Adrian Shuttleworth, Cay M Kielty

**Affiliations:** Wellcome Trust Centre for Cell-Matrix Research, Faculty of Life Sciences, University of Manchester, Manchester, United Kingdom

**Keywords:** Adult stem cells, Multipotential differentiation, Mesenchymal stem cells, Marrow stromal stem cells

## Abstract

Defining the signaling mechanisms that regulate the fate of adult stem cells is an essential step toward their use in regenerative medicine. Platelet-derived growth factor receptor (PDGFR) signaling plays a crucial role in specifying mesenchymal stem cell (MSC) commitment to mesenchymal lineages. Based on the hypothesis that selective inhibition of signaling pathways involved in differentiation may increase stem cell potency, we examined the role of PDGFR signaling in controlling the fate of human MSCs. Using a small molecular PDGFR inhibitor that induced MSCs toward a more rounded shape, expression of Oct4 and Nanog were markedly upregulated. In these PDGFR inhibitor-treated MSCs, Oct4 and Nanog expression and cell shape were regulated by janus kinase (JAK), MAPK kinase (MEK), and epidermal growth factor receptor (EGFR) signaling. Under defined differentiation conditions, these PDGFR-inhibited MSCs expressed definitive endodermal, ectodermal, and mesodermal markers. We also confirmed that depletion of individual PDGF receptors upregulated expression of Oct4A and Nanog. This study identifies PDGFR signaling as a key regulator of Oct4 and Nanog expression and of MSC potency. Thus, inhibiting these specific receptor tyrosine kinases, which play essential roles in tissue formation, offers a novel approach to unlock the therapeutic capacity of MSCs. STEM CELLS *2012;30:548–560*

## INTRODUCTION

A fundamental step toward using adult stems cells for tissue regeneration is identifying signaling mechanisms that regulate their fate. Bone marrow-derived mesenchymal stem cells (MSCs), also known as multipotent stromal cells [1], are therapeutically appealing since they are readily isolated and expanded in culture [2, 3] and exhibit immunosuppressive and anti-inflammatory properties [4, 5]. However, their differentiation is generally limited to mesenchymal lineages such as osteocytes, chondrocytes, and adipocytes [3].

Recent reports have demonstrated the efficacy of regulating stem cell fate with small molecular inhibitory compounds which target signaling pathways implicated in directing differentiation or maintaining pluripotency. These studies have primarily focused on controlling embryonic stem cell (ESC) pluripotency or differentiation [6] or modulating somatic cell reprogramming to generate induced pluripotent stem cells [7]. There is however a paucity of information on signaling pathways which can be targeted to regulate MSC multipotency.

MSCs express abundant platelet-derived growth factor receptors (PDGFRs, PDGFRα and PDGFRβ) [8], which play a crucial role in specifying their commitment to osteogenic, chondrogenic, or adipogenic fates. While both PDGFRs can activate the same phosphoinositide 3-kinase (PI3K), PLCγ, and mitogen-activated protein kinase (MAPK) signaling pathways, each receptor can induce distinct cellular responses [10]. Transcriptional profiling revealed that, of the pathways identified as being important in MSC differentiation, PDGF was more significant than fibroblast growth factor (FGF) or transforming growth factor (TGF)-β signaling [9]. MSC differentiation is also dictated by cell shape [11], which is governed by actomyosin tension. PDGFR signaling directly controls cytoskeletal actin reorganization and actomyosin-mediated contractility [8, 12] and can activate cAbl [13] that also regulates actin reorganization [14].

The embryonic transcription factors Oct4 and Nanog are crucial for specifying the pluripotent status of ESCs [15]. Nuclear-located Oct4A is responsible for regulating pluripotency, while the Oct4B isoform, which is generally expressed in the cytoplasm, cannot sustain stem cell properties [16]. Previous studies have reported the variable expression of Oct4 and Nanog by MSCs, which was dependent on the culture conditions [17, 18]. The expression of Oct4 in MSCs was shown to target similar genes to those in ESCs [19] and increased differentiation toward osteocytes and adipocytes [18]. However, the mechanisms regulating Oct4 and Nanog expression in MSCs remain unknown.

Based on the premise that selective inhibition of signaling pathways involved in MSC differentiation may enhance multipotency, we used a small molecular inhibitor [20] to block PDGFR and downstream cAbl signaling, which induced a more rounded MSC shape. This inhibition produced a pronounced increase in Oct4 and Nanog levels, which was regulated by janus kinase (JAK)-signal transducer and activator of transcription 3 (STAT3) signaling and actomyosin contractility. These MSCs were induced to express definitive markers for ectoderm, endoderm, and mesoderm lineages, demonstrating their increased multipotency. This study demonstrates that inhibition of PDGFR signaling is an important regulator of Oct4 and Nanog expression and of MSC potency.

## MATERIALS AND METHODS

### Cell Culture

Human MSCs from bone marrow of 21- and 26-year old females and 19- and 33-year old males (Lonza, Lonza Cologne GmbH, Germany, http://www.lonzabio.com/) were cultured on 0.1% gelatin and maintained in MesenPRO RS basal medium (Invitrogen, Invitrogen, NY 14072, USA, http://www.invitrogen.com/) and analyzed at passage 5. Prior to analysis, MSCs were cultured in Knockout Dulbecco's modified Eagle's medium, containing 15% Knockout SR (Invitrogen) (ESC medium) for 24 hours.

### Small Molecular Inhibitors

All the molecular inhibitors used in this study were obtained from Merck, Merck Chemicals, Nottingham, UK, http://www.merck-chemicals.co.uk/. They were PDGFR inhibitor-IV (#521233), PDGFR inhibitor-V (#521234), epidermal growth factor receptor (EGFR) (PD168393), fibroblast growth factor receptor (FGFR) (341608), MAPK kinase (MEK) (PD98059), PI3K (LY294002), STAT3 (Inhibitor VI), glycogen synthase kinase (GSK)-3 (Inhibitor IX), JAK (Inhibitor I), Rho-kinase (H-1152), Blebbistatin (#203391), and Latrunculin B (#428020). Further details of these inhibitors are given in Supporting Information Table 1.

### PCR and quantitative PCR

Total RNA was isolated using Trizol reagent (Invitrogen) followed by digestion with RNase-free DNase (Promega, Promega UK, Hampshire, UK, http://www.promega.com/). First strand cDNA synthesis was performed using avian myeloblastosis virus (AMV) reverse transcriptase (Roche, Roche UK, Welwyn Garden City, UK, http://www.roche.co.uk/), PCR using expand high fidelity PCR system (Roche), and real-time PCR using SYBR green quantitative PCR core kit (Eurogentec). Gene expression was determined relative to glyceraldehyde 3-phosphate dehydrogenase (GAPDH) using the ΔC_t_ method. All primer sequences are provided in Supporting Information Table 2.

### siRNA Knockdown

MSCs were transfected with small interfering RNAs (siRNAs) by electroporation using a human Nucleofector kit (Lonza), allowed to adhere in growth medium, and then cultured overnight in ESC medium. Validated siRNAs functionally tested to provide ≥70% target gene knockdown were used to transiently knockdown PDGFRA (SI02659699) or PDGFRB (SI00605745) (Qiagen, Qiagen UK, Horsham, UK, http://www.qiagen.co.uk/), and two different siRNAs were used to knockdown ABL1 (4390824) (Ambion, Ambion, Paisley, UK, http://www.invitrogen.com/) (SI00299103) (Qiagen). Scrambled siRNA (Qiagen) was used as a control.

### Immunoblotting

Protein isolation and immunoblotting were performed as previously described [8]. Isolation of nuclear and cytoplasmic extracts was performed using a nuclear extraction kit (Active Motif). Details of the antibodies used are given in Supporting Information Table 3. Protein quantitation was determined using Gene Tools Software (Syngene, Syngene UK, Cambridge, UK, http://www.syngene.com/syngene-uk/).

### Immunofluorescence Microscopy

Immunofluorescence was performed as previously described [21], except cells were permeabilized using ice-cold methanol for STAT3 (Y705). Details of the antibodies used are given in Supporting Information Table 3. Images were collected on a Nikon C1 confocal using a TE2000 PSF inverted microscope, using 60× /NA 1.40 Plan Apo or 20× /NA 0.50 Plan Fluor objectives and 3× confocal zoom. Different sample images detecting the same antibodies were acquired under constant acquisition settings. Images were processed using Nikon EZ-C1 FreeViewer v3.3 software. Bright-field images were collected on an Olympus BX51 widefield microscope, using a 10× /NA 0.3 UPlan F1 objective. Images were captured with a CoolSNAP camera system and processed using MetaMorph imaging v5.0 software.

### Cell Image Analysis

MSC size and shape were measured using CellProfiler image analysis vr10997 software [22] using a pipeline for human cells. Analysis was performed from images obtained using a Nikon C1 confocal microscope and 20× objective, with nuclei identified by 4′,6-diamino-2-phenylindole (DAPI) staining and cells identified by wheat germ agglutinin and phalloidin staining. Cells touching the edge of the image were excluded from analysis.

### Proteome Arrays and Immunoassays

A human pluripotent stem cell array kit (ARY010) or phospho receptor tyrosine kinase array kit (RTK) (ARY001) (R&D Systems, R&D Systems Europe Ltd., http://www.RnDSystems.co.uk) was used to simultaneously determine the relative expression levels of 15 different stem cell markers or phosphorylation levels of 42 different RTKs, respectively. PDGFR immunoassays were performed as previously described [21].

## RESULTS

### PDGFR Inhibitor-IV Increased Oct4 and Nanog Expression

**Figure 1:**
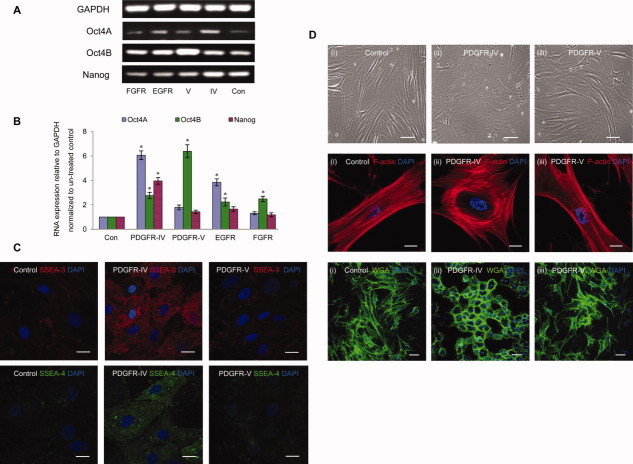
PDGFR inhibitor regulated Oct4 and Nanog expression and mesenchymal stem cell (MSC) morphology. (A): RT-PCR analysis examining Oct4A, Oct4B, and Nanog expression, with GAPDH as loading controls, following 24-hour exposure to 0.1 μM PDGFR inhibitor-IV, 20 nM PDGFR inhibitor-V, 2 nM EGFR, and 0.1 μM FGFR inhibitors. (B): Quantitative RT-PCR analysis determining Oct4A, Oct4B, and Nanog expression relative to GAPDH and normalized to untreated controls, following 24-hour exposure to 0.1 μM PDGFR inhibitor-IV, 20 nM PDGFR inhibitor-V, 2 nM EGFR, and 0.1 μM FGFR inhibitors. *, *p* < .001 compared with untreated controls, using paired *t*-test *n* > 3 separate experiments, error bars represent SD. (C): Immunofluorescence analysis examining SSEA-3 and SSEA-4 expression, following 24-hour exposure to 0.1 μM PDGFR inhibitor-IV, 20 nM PDGFR inhibitor-V, or untreated controls. Representative images show SSEA-3 (red), SSEA-4 (green), and nuclei (blue). Scale bars = 20 μm. (D): Representative bright-field and immunofluorescence images of MSC morphology, (i) untreated controls or following 24-hour exposure to (ii) 0.1 μM PDGFR inhibitor-IV, or (iii) 20 nM PDGFR inhibitor-V. Fluorescence images show F-actin filaments (red), wheat germ agglutinin (green), and nuclei (blue). Scale bars = 100 μm (bright-field images) and 20 μm (immunofluorescence images). Abbreviations: DAPI, 4′,6-diamino-2-phenylindole; EGFR, epidermal growth factor receptor; FGFR, fibroblast growth factor receptor; GAPDH, glyceraldehyde 3-phosphate dehydrogenase; PDGFR, platelet-derived growth factor receptor; RT-PCR, reverse transcription polymerase chain reaction; SSEA, stage-specific embryonic antigen; WGA, wheat germ agglutinin.

To investigate how PDGFR signaling may influence MSC potency, the effects of two cell-permeable small molecular inhibitory compounds, PDGFR inhibitor-IV [20] and PDGFR inhibitor-V [23], on the expression of the pluripotent transcription factors Oct4A and Nanog were determined (Fig. 1A). Since epidermal growth factor (EGF) and FGF receptors may also contribute in regulating MSC differentiation [9, 24], small molecular inhibitory compounds to block EGF or FGF receptor activity were also tested.

Reverse transcription polymerase chain reaction (RT-PCR) (Fig. 1A) and quantitative RT-PCR (Fig. 1B) demonstrated that, of the inhibitory compounds examined, exposure to PDGFR inhibitor-IV for 24 hours produced the greatest increase in both Oct4A (6.1 ± 0.4-fold) and Nanog (4.0 ± 0.3-fold) transcripts. In comparison, PDGFR inhibitor-V (Oct4A, 1.8 ± 0.2-fold; Nanog, 1.4 ± 0.2-fold), EGFR inhibitor (Oct4A, 3.8 ± 0.3-fold; Nanog, 1.7 ± 0.2-fold), and FGFR inhibitor (Oct4A, 1.3 ± 0.1-fold; Nanog, 1.2 ± 0.2-fold) all induced lower expression levels (Fig. 1B). To specifically identify the Oct4A transcript, the 5′-primer sequence incorporated a unique polymorphism [25]. Whereas PDGFR inhibitor-V also markedly increased Oct4B expression (6.4 ± 0.5-fold), PDGFR inhibitor-IV induced a much lower level of Oct4B expression (2.8 ± 0.3-fold). Thus, PDGFR inhibitor-IV preferentially upregulated Oct4A. We also showed that PDGFR inhibitor-IV had a minimal effect on the prominent basal EGFR activity of MSCs (Supporting Information Fig. S1C).

The expression of stage-specific embryonic antigens (SSEA-3 and SSEA-4) was also examined. Immunofluorescence analysis demonstrated that, compared to control MSCs and those exposed to PDGFR inhibitor-V, treatment with PDGFR inhibitor-IV for 24 hours induced SSEA4 and particularly SSEA3 expression (Fig. 1C).

We confirmed that phosphorylation levels of PDGFRα and PDGFRβ were suppressed by both PDGFR inhibitor-IV (Supporting Information Fig. S1A, S1C) and PDGFR inhibitor-V (Supporting Information Fig. S1B). An important distinction between the two compounds is that PDGFR inhibitor-IV also inhibits cAbl activity (IC_50_ = 22 nM), while PDGFR inhibitor-V has little or no effect on cAbl (IC_50_ > 1 μM) (Supporting Information Table 1). These differential effects on cAbl phosphorylation were also confirmed (Supporting Information Fig. S1D), including the efficiency of PDGFR inhibitor-IV in suppressing nuclear cAbl phosphorylation (Supporting Information Fig. S1E). These results demonstrated that the combined inhibitory effects of PDGFR inhibitor-IV on PDGFR and cAbl signaling upregulated Oct4 and Nanog expression.

### PDGFR Inhibitor-IV Induced an MSC Shape Change

Signaling through PDGFRs, EGFRs, and integrins has been shown to activate cAbl phosphorylation [26]. Cytoplasmic cAbl controls actin filament rearrangement and thus regulates cell shape [14]. Examination of MSC morphology by phalloidin or wheat germ agglutinin staining to detect intracellular actin filaments or cell surface lectin expression, respectively, demonstrated that untreated control MSCs and those exposed to PDGFR inhibitor-V had an elongated shape, whereas PDGFR inhibitor-IV induced a more rounded shape (Fig. 1D). In contrast, MSCs exposed to EGFR inhibitor (Fig. 4F) or FGFR inhibitor retained their elongated morphology (data not shown), indicating that cAbl signaling through these receptors had little effect on MSC shape.

These results demonstrated that the inhibitory effects of PDGFR inhibitor-IV on PDGFR and cAbl signaling induced a prominent change in MSC shape and actin filament organization.

### PDGFR Inhibitor-IV Increased the Nucleus/Cytoplasm Ratio

**Figure 2:**
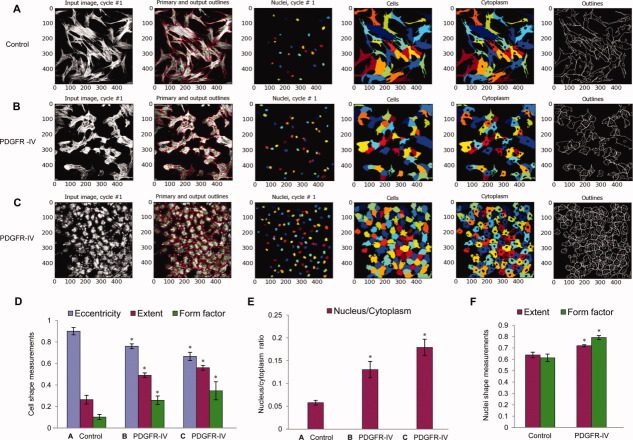
PDGFR inhibitor produced a significant change in mesenchymal stem cell (MSC) shape. (A–C): Cell image processing to determine the size and shape measurements of MSCs: (A) untreated controls at low density, (B) MSCs at low density exposed to 0.1 μM PDGFR inhibitor-IV for 24 hours, and (C) MSCs at high density exposed to 0.1 μM PDGFR inhibitor-IV for 24 hours. Representative input image with the processed primary and output outlines, nuclei, cells, cytoplasm, and shape outlines are shown. (D): Representative cell shape measurements (eccentricity, extent, and form factor) determined from six different processed images of (A) untreated control MSCs at low density, (B) MSCs at low density, or (C) MSCs at high density exposed to 0.1 μM PDGFR inhibitor-IV for 24 hours. *, *p* < .001 compared with untreated controls, using paired *t* test *n* = 6 different images from two separate experiments; error bars represent SD. (E): Representative nucleus/cytoplasm ratio measurements determined from six different processed images of (A) untreated control MSCs at low density, (B) MSCs at low density, or (C) MSCs at high density exposed to 0.1 μM PDGFR inhibitor-IV for 24 hours. *, *p* < .001 compared with untreated controls, using paired *t* test *n* = 6 different images from two separate experiments, error bars represent SD. (F): Representative nuclei shape measurements (extent and form factor) determined from six different processed images of MSCs at low density, untreated controls or exposed to 0.1 μM PDGFR inhibitor-IV for 24 hours. *, *p* < .001 compared with untreated controls, using paired *t*-test *n* = 6 different images from two separate experiments; error bars represent SD. Abbreviation: PDGFR, platelet-derived growth factor receptor.

To further examine the effects of PDGFR inhibitor-IV on MSC shape, we used cell image analysis software (CellProfiler) to quantitate size and shape measurements for every cell within an input image [22]. Since cell density can affect cell shape [11], image analysis of untreated control MSCs at low density (Fig. 2A), MSCs at low density exposed to PDGFR inhibitor-IV (Fig. 2B), and MSCs at higher density exposed to PDGFR inhibitor-IV (Fig. 2C) were processed. The actual data derived from the input images of Figures 2A and 2B are shown in Supporting Information Table 4. Three different shape features were used for quantitation: eccentricity of an ellipse (circle = 0, line = 1), extent, and form factor (1 = circle). Compared with untreated controls (eccentricity 0.93 ± 0.03; extent 0.26 ± 0.04; form factor 0.10 ± 0.02), MSCs of a similar density exposed to PDGFR inhibitor-IV (eccentricity 0.76 ± 0.02; extent 0.49 ± 0.02; form factor 0.26 ± 0.04) adopted a more rounded shape (Fig. 2D). Similarly, MSCs at a higher density exposed to PDGFR inhibitor-IV (eccentricity 0.66 ± 0.03; extent 0.56 ± 0.02; form factor 0.35 ± 0.08) had increased circularity (Fig. 2D). Measurements of the areas of nuclei and cytoplasm also revealed that, compared with the nucleus/cytoplasm ratio of controls (0.057 ± 0.005), PDGFR inhibitor-IV-treated MSCs at a similar density or higher density had significantly higher ratios (0.134 ± 0.018 and 0.183 ± 0.018, respectively) (Fig. 2E). Furthermore, nuclei shape measurements (extent and form factor) revealed that PDGFR inhibitor-IV-treated MSCs had a significantly more rounded nuclei than controls (Fig. 2F). Thus PDGFR inhibitor-IV not only induced MSCs to become more rounded but also changed their nuclei shape and increased the nucleus/cytoplasm ratio.

### PDGFRα, PDGFRβ, or cAbl Knockdown Increased Oct4 and Nanog Expression

**Figure 3:**
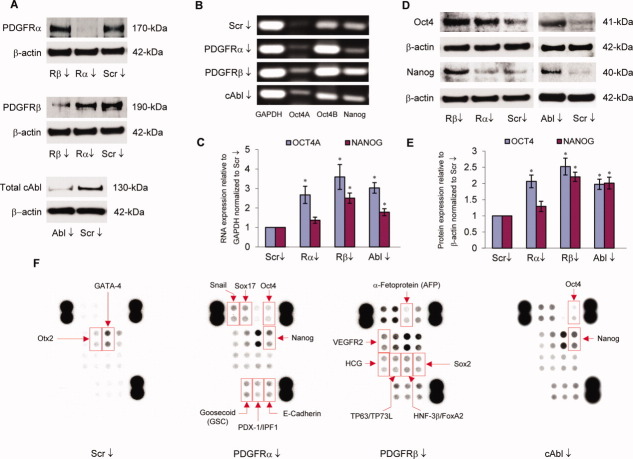
Knockdown of PDGFRs or cAbl upregulated Oct4 and Nanog. (A): Immunoblot analysis of PDGFRα, PDGFRβ, or cAbl expression, with β-actin as a loading control, following corresponding small interfering RNA (siRNA)-mediated knockdown. (B): RT-PCR analysis examining Oct4A, Oct4B, and Nanog expression, with GAPDH as a loading control, following siRNA-mediated PDGFRα, PDGFRβ, or cAbl knockdown. (C): Quantitative RT-PCR determining Oct4A and Nanog transcript levels relative to GAPDH and normalized to Scr control knockdowns, following siRNA-mediated PDGFRα, PDGFRβ, or cAbl knockdown. *, *p* < .001 compared with siRNA Scr control knockdowns, using paired *t* test *n* > 3 separate experiments; error bars represent SD. (D): Immunoblot analysis examining Oct4 and Nanog expression with β-actin as loading controls, following siRNA-mediated PDGFRα, PDGFRβ, or cAbl knockdown, with siRNA Scr control knockdown as control. (E): Relative protein expression levels, normalized to Scr control knockdowns. *, *p* < .001 compared with siRNA Scr control knockdowns, using paired *t* test *n* > 3 separate experiments; error bars represent SD. (F): Proteome array analysis to determine the relative expression levels of mesoderm, endoderm, and pluripotent markers, following siRNA-mediated PDGFRα, PDGFRβ, or cAbl knockdown, with siRNA Scr control knockdown as control. Each of the array markers has duplicate spots, which are boxed to highlight their identity. Abbreviations: AFP, α-fetoprotein; GAPDH, glyceraldehyde 3-phosphate dehydrogenase; GSC, goosecoid; HCG, human chorionic gonadotropin; HNF, hepatocyte nuclear factor; PDGFR, platelet-derived growth factor receptor; RT-PCR, reverse transcription polymerase chain reaction; Scr, scrambled; VEGFR, vascular endothelial growth factor receptor.

The contributions of PDGFRs and cAbl to regulate Oct4 and Nanog expression was further examined by PDGFRα, PDGFRβ, or cAbl knockdown. Compared with control scrambled siRNA-treated MSCs, PDGFRα knockdown ablated PDGFRα protein expression but had minimal effect on PDGFRβ protein, while PDGFRβ knockdown markedly reduced PDGFRβ protein expression, with no detectable impact on PDGFRα protein (Fig. 3A). Therefore, PDGFRα and PDGFRβ siRNAs demonstrated target knockdown efficiency and specificity between PDGFRs. Two different cAbl siRNAs were shown to suppress cAbl protein expression (Fig. 3A). The effect of each individual knockdown on MSC morphology after 24 hours was minimal (data not shown).

RT-PCR (Fig. 3B) and quantitative RT-PCR (Fig. 3C) demonstrated that while PDGFRα knockdown increased Oct4A (2.7 ± 0.4-fold) and Nanog (1.4 ± 0.2-fold), PDGFRβ or cAbl knockdown produced a higher level of Oct4A (3.6 ± 0.6-fold and 3.0 ± 0.3-fold, respectively) and Nanog (2.5 ± 0.3-fold and 1.8 ± 0.2-fold respectively). PDGFRα or PDGFRβ knockdowns also increased Oct4B, but cAbl knockdown had less effect on Oct4B expression (Fig. 3B), suggesting that cAbl knockdown preferentially increased the Oct4A isoform.

Immunoblot analysis, using an Oct4 antibody recognizing a single epitope within Oct4A [25], showed that PDGFRα knockdown increased Oct4 (2.1 ± 0.2-fold), but Nanog expression remained virtually unchanged (1.2 ± 0.3-fold) (Fig. 2D, 2E). However, PDGFRβ or cAbl knockdown each increased the expression levels of Oct4 (2.6 ± 0.3-fold and 2.0 ± 0.2-fold, respectively) and Nanog (2.2 ± 0.1-fold and 2.0 ± 0.2-fold, respectively) (Fig. 3D, 3E). These results demonstrate that the PDGFR inhibitor-IV-induced increase in Oct4 and Nanog expression is primarily mediated by blocking PDGFRβ and cAbl signaling.

Following PDGFRα, PDGFRβ, or cAbl knockdowns, equal concentrations of individual lysates were further analyzed using a human pluripotency marker stem cell array to simultaneously detect the relative expression levels of 15 different stem cell markers. In comparison to scrambled siRNA-treated MSCs, PDGFRα knockdown upregulated virtually all the pluripotency markers. Notably, PDGFRα knockdown increased mesoderm (Goosecoid, Snail), endoderm (Sox17, E-cadherin) markers, and Oct4 (Fig. 3F). PDGFRβ knockdown resulted in even higher levels of the proteome markers, especially Snail, Sox17, VEGFR2, Oct4, and Nanog (Fig. 3F). Knockdown of cAbl was similar to PDGFRα; however, Nanog expression was higher (Fig. 3F). Thus, each individual knockdown of PDGFRα, PDGFRβ, or cAbl increased mesoderm and endoderm markers; the PDGFRβ or cAbl knockdowns also increased Oct4 and Nanog, producing an expression profile similar to mesendoderm.

### PDGFR Inhibitor-IV-Induced Oct4 and Nanog Expression Was STAT3 Dependent

**Figure 4:**
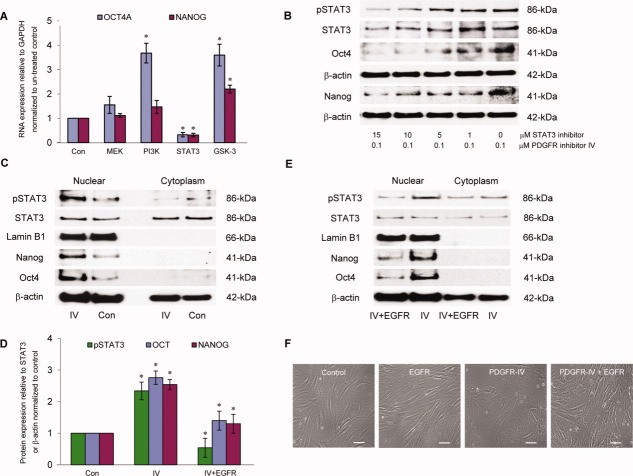
PDGFR inhibition regulated STAT3 signaling and nuclear translocation. (A): Quantitative RT-PCR analysis examining Oct4A and Nanog transcript levels relative to GAPDH and normalized to untreated controls, following 24-hour exposure to 20 μM MEK, 5 μM PI3K, 10 μM STAT3, or 10 nM GSK-3 inhibitors. *, *p* < .001 compared with untreated controls, using paired *t* test; *n* > 3 separate experiments, error bars represent SD. (B): Immunoblot analysis of Oct4 and Nanog with β-actin as loading controls, and STAT3 (Y705) and total STAT3 expression levels, following exposure to 0.1 μM PDGFR inhibitor-IV and an increasing dose of STAT3 inhibitor (0–15 μM) for 24 hours. (C): Immunoblot analysis examining nuclear and cytoplasm expression of pSTAT3 (Y705), Oct4, and Nanog, with total STAT3 and β-actin as corresponding loading controls, and nuclear membrane marker Lamin B1, following 24 hours exposure to 0.1 μM PDGFR inhibitor-IV or untreated controls. (D): Relative protein expression levels in nuclear extracts, normalized to untreated controls. *, *p* < .001 for (IV) treatment or (IV+EGFR) treatment compared with untreated controls, using paired *t* test; *n* > 3 separate experiments; error bars represent SD. (E): Immunoblot analysis examining nuclear and cytoplasm expression of pSTAT3 (Y705), Oct4, and Nanog, with total STAT3 and β-actin as corresponding loading controls, and nuclear membrane marker Lamin B1, following 24 hours exposure to 0.1 μM PDGFR inhibitor-IV alone or in the presence of 2 nM EGFR (EGFR inhibitor). (F): Representative bright-field images of control mesenchymal stem cell morphology and following 24-hour exposure to 2 nM EGFR, 0.1 μM PDGFR inhibitor-IV, or 0.1 μM PDGFR inhibitor-IV with 2 nM EGFR. Scale bar = 100 μm. Abbreviations: EGFR, epidermal growth factor receptor; GAPDH, glyceraldehyde 3-phosphate dehydrogenase; GSK, glycogen synthase kinase; MEK, MAPK kinase; PDGFR, platelet-derived growth factor receptor; PI3K, phosphoinositide 3-kinase; RT-PCR, reverse transcription polymerase chain reaction; STAT, signal transducer and activator of transcription.

Having established the critical role of PDGFRs and cAbl signaling in regulating Oct4 and Nanog expression, we went on to identify other signaling pathways involved. Small molecular inhibitors were used to target four different signaling pathways implicated in regulating ESC pluripotency: MAPK-extracellular signal-regulated kinase (ERK), PI3K, STAT3, and Wnt [27]. Quantitative RT-PCR demonstrated that inhibition of PI3K significantly increased Oct4A expression (3.6 ± 0.4-fold), and inhibition of GSK-3 to activate Wnt signaling increased both Oct4A (3.6 ± 0.5-fold) and Nanog (2.2 ± 0.2-fold) expression, whereas inhibition of MEK to suppress ERK signaling had no significant effect (Fig. 4A). In contrast, the STAT3 inhibitor markedly decreased Oct4A and Nanog expression (3.3 ± 0.3-fold and 3.3 ± 0.2-fold decrease, respectively) (Fig. 4A); therefore, the involvement of the JAK-STAT3 pathway in regulating Oct4 and Nanog was evaluated further.

Cytokine receptors, tyrosine kinase receptors including PDGFRβ and EGFR, as well as nonreceptor tyrosine kinases, including cAbl, are known to activate STAT3 signaling [28, 29], which plays a pivotal role in inducing pluripotency [30]. To determine whether STAT3 signaling was involved in mediating PDGFR inhibitor-IV-induced Oct4 and Nanog expression, the effect of increasing inhibition of STAT3 in the presence of PDGFR inhibitor-IV was examined. Immunoblot analysis demonstrated that an increasing dose of STAT3 inhibitor produced a proportional decrease in PDGFR inhibitor-IV-induced Oct4 and a marked reduction in Nanog expression (Fig. 4B). The same analysis confirmed the effectiveness of the STAT3 inhibitor in decreasing STAT3 (Y705) phosphorylation (Fig. 4B). Thus, STAT3 signaling is essential for PDGFR inhibitor-IV-induced Oct4 and Nanog expression.

Immunoblot analysis of nuclear and cytoplasmic extracts demonstrated that, compared with untreated controls, PDGFR inhibitor-IV increased the level of nuclear localized Oct4 (2.8 ± 0.3-fold), Nanog (2.5 ± 0.2-fold), and STAT3 (Y705) (2.3 ± 0.3-fold) (Fig. 4C, 4D) and increased the STAT3 (Y705) nuclear/cytoplasm ratio (2.6 ± 0.5-fold). Immunofluorescence analysis also demonstrated that MSCs exposed to PDGFR inhibitor-IV displayed an increase in the STAT3 (Y705) nuclear/cytoplasm ratio (2.9 ± 0.3-fold) (Supporting Information Fig. S2A, S2B). Thus exposure to PDGFR inhibitor-IV not only increased the expression of nuclear Oct4 and Nanog but also enhanced the nuclear translocation of STAT3 (Y705). Interestingly, another PDGFR and cAbl inhibitor, imatinib, has also been shown to induce sustained activation of STAT3 [31].

### PDGFR Inhibitor-IV-Induced Cell Rounding Was Dependent on Basal EGFR Activity

Since the PDGFR inhibitor-IV did not block EGFR basal activity (Supporting Information Fig. S1C), we investigated whether basal EGFR activity in the presence of the PDGFR inhibitor-IV contributes to cell shape change and STAT3 (Y705) nuclear translocation. Immunoblot analysis of nuclear and cytoplasmic extracts demonstrated that, compared with MSCs treated with PDGFR inhibitor-IV only, cells exposed to both PDGFR inhibitor-IV and EGFR inhibitor decreased expression of nuclear Oct4 (2.4 ± 0.3-fold), Nanog (2.3 ± 0.3-fold), and STAT3 (Y705) (2.8 ± 0.4-fold) (Fig. 4D, 4E) and reduced the STAT3 (Y705) nuclear/cytoplasm ratio (2.3 ± 0.4-fold). Furthermore, EGFR inhibition in the presence of PDGFR inhibitor-IV also “reversed” the distinctive PDGFR inhibitor-IV-induced MSC shape back toward a more elongated morphology (Fig. 4F). Thus in the presence of PDGFR inhibitor-IV, basal EGFR signaling (cAbl-independent in the presence of this inhibitor) contributes to MSC rounding, increased nuclear STAT3 (Y705), and increased Oct4 and Nanog expression. The ablation of PDGFR signaling may enable basal EGFR signaling to increase nuclear STAT (Y705) through mechanisms that also influence cell shape (Discussion section).

We also examined the consequences of directly stimulating EGFR on nuclear STAT3 and on Oct4 and Nanog expression. While MSCs exposed to EGF demonstrated increased nuclear STAT3 (Y705) translocation (data not shown), quantitative RT-PCR showed minimal effect on Oct4A and Nanog expression (Supporting Information Fig. S2C). Thus increased nuclear STAT3 (Y705) alone is insufficient to induce Oct4 and Nanog expression when PDGFRs are not inhibited.

### PDGFR Inhibition-Induced MSC Shape Change Was MEK and JAK Dependent

**Figure 5:**
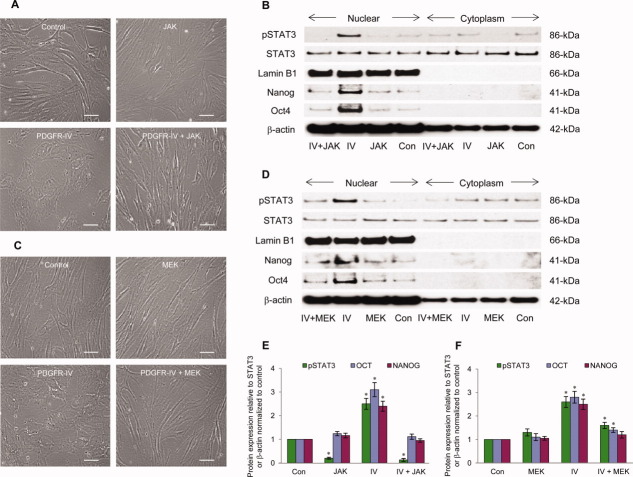
PDGFR inhibitor-induced mesenchymal stem cell (MSC) shape change was regulated by MEK and JAK. (A): Representative bright-field images of MSC morphology and (B) immunoblot analysis examining nuclear and cytoplasm expression of STAT3 (Y705), Oct4, and Nanog with total STAT3 and β-actin as corresponding loading controls and nuclear membrane marker Lamin B1, following 24 hours exposure to 0.1 μM PDGFR inhibitor-IV, 20 nM JAK inhibitor, 0.1 μM PDGFR inhibitor-IV together with 20 nM JAK inhibitor, or untreated controls. Scale bars = 100 μm. (C): Representative bright-field images of MSC morphology and (D) immunoblot analysis examining nuclear and cytoplasm expression of STAT3 (Y705), Oct4, and Nanog with total STAT3 and β-actin as corresponding loading controls and nuclear membrane marker Lamin B1, following 24 hours exposure to 0.1 μM PDGFR inhibitor-IV, 20 μM MEK inhibitor, 0.1 μM PDGFR inhibitor-IV, and 20 μM MEK inhibitor, or untreated controls. Scale bars = 100 μm. (E, F): Relative protein expression levels in nuclear extracts, normalized to untreated controls. *, *p* < .001 untreated controls, using paired *t* test *n* > 3 separate experiments; error bars represent SD. Abbreviations: JAK, janus kinase; PDGFR, platelet-derived growth factor receptor; MEK, MAPK kinase; STAT, signal transducer and activator of transcription.

Cytoskeletal actin filaments that modulate cell morphology are regulated by the Rho family of small GTPases: RhoA, Rac1, and Cdc42 [32]. More recently, active RhoA, Rac1, and Cdc42 have all been shown to regulate STAT3 phosphorylation and nuclear translocation [33], while STAT3 can also regulate Rac1 activity, actin reorganization, and actomyosin contractility [34]. We therefore investigated the involvement of JAK-STAT3 signaling in regulating the MSC shape. While JAK inhibition effectively blocked STAT3 (Y705) (Supporting Information Fig. S2D), there was no detectable effect on MSC morphology (Fig. 5A). However, JAK inhibition changed the distinctive PDGFR inhibitor-IV-induced rounded MSC shape to a more elongated morphology (Fig. 5A).

Immunoblot analysis of nuclear and cytoplasmic extracts demonstrated that this JAK inhibition-induced MSC shape change was accompanied by a decrease in nuclear Oct4 (2.8 ± 0.4-fold), Nanog (2.6 ± 0.3-fold), and STAT3 (Y705) (19.2 ± 0.8-fold) (Fig. 5B, 5E) and reduced the STAT3 (Y705) nuclear/cytoplasm ratio (3.2 ± 0.2-fold) (Fig. 5B). In contrast, JAK inhibition alone had little effect on Oct4 and Nanog expression (Fig. 5B), further demonstrating that nuclear STAT3 alone does not regulate Oct4 and Nanog.

MEK-ERK signaling can regulate the level of STAT3 (Y705) and its nuclear translocation [35]. Moreover, active MEK can downregulate Rho-associated kinase (ROCK) activity, decreases actin stress fiber assembly and actomyosin contractility, while MEK inhibition restores ROCK activity [36]. We therefore examined the effect of MEK inhibition on MSC morphology. While MEK inhibition effectively suppressed ERK1/2 phosphorylation (Supporting Information Fig. S2E), it had no detectable effect on MSC morphology (Fig. 5C). However, MEK inhibition reversed the PDGFR inhibitor-IV MSC shape, restoring a similar morphology to controls (Fig. 5C).

Immunoblot analysis of nuclear and cytoplasmic extracts demonstrated that this MEK inhibition-induced MSC shape change was accompanied by a decrease in nuclear Oct4 (2.0 ± 0.2-fold), Nanog (2.1 ± 0.3-fold), and STAT3 (Y705) (1.6 ± 0.2-fold) (Fig. 5D, 5F) and reduced the STAT3 (Y705) nuclear/cytoplasm ratio (2.1 ± 0.4-fold) (Fig. 5D). Taken together, the results demonstrate that the distinctive PDGFR inhibitor-IV-induced rounded MSC shape is JAK-STAT3 and MEK-ERK signaling dependent.

### Decreased Actomyosin Tension Regulated Oct4, Nanog, and STAT3 (Y705)

To further investigate how MSC shape may regulate Oct4 and Nanog expression, we examined the effects of decreasing actomyosin contractility, by exposing MSCs to an increasing dose of ROCK inhibitor H-1152, Blebbistatin that inhibits myosin II ATPase activity, or Latrunculin B that inhibits actin filament polymerization.

**Figure 6:**
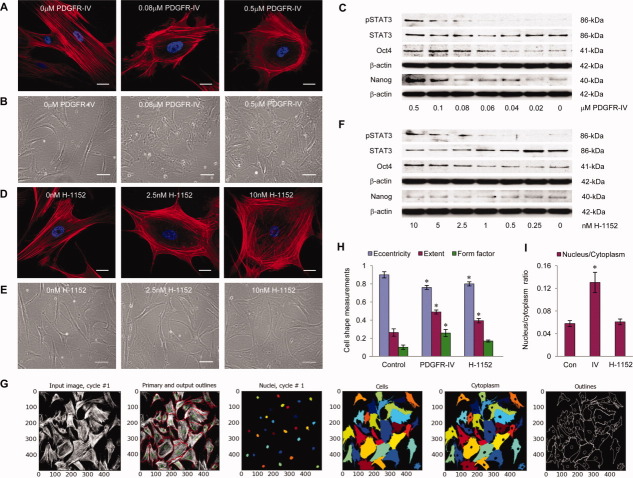
Rho-associated kinase inhibition induced a mesenchymal stem cell (MSC) shape change and increased Oct4 and STAT3 (Y705). (A, D): Immunofluorescence analysis examining F-actin filament organization and MSC shape, following 24-hour exposure to an increasing dose of (A) PDGFR inhibitor-IV (0–0.5 μM) or (D) H-1152 (0–10 nM). Representative images show F-actin filaments (red) and nuclei (blue). Scale bars = 20 μm. (B, E): Representative bright-field images of MSC morphology following 24 hours exposure to an increasing dose of (B) PDGFR inhibitor-IV (0–0.5 μM) or (E) H-1152 (0-10 nM). Scale bars = 100 μm. (C, F): Immunoblot analysis determining Oct4, Nanog, and pSTAT3 (Y705) expression, with β-actin and total STAT3 as corresponding loading controls, following 24-hour exposure to an increasing dose of (C) PDGFR inhibitor-IV (0–0.5 μM) or (F) H-1152 (0–10 nM). (G): Image processing to determine the size and shape measurements of MSCs, following 24-hour exposure to 5 nM H-1152. A representative input image with the processed primary and output outlines, nuclei, cells, cytoplasm and shape outlines, is shown. (H): Representative shape measurements (eccentricity, extent, and form factor) determined from six different processed images of untreated control MSCs, or following 24 hours exposure to 0.1 μM PDGFR inhibitor-IV or 5 nM H-1152. (I): Representative nucleus/cytoplasm ratio measurements determined from six different processed images of untreated control MSCs, or following 24 hours exposure to 0.1 μM PDGFR inhibitor-IV or 5 nM H-1152. Abbreviations: PDGFR, platelet-derived growth factor receptor; STAT, signal transducer and activator of transcription.

We first examined the effects of using an increasing dose of PDGFR inhibitor-IV. Immunofluorescence analysis confirmed that as PDGFR inhibition increased, MSCs became more rounded having concentric rings of actin filaments around the cell periphery (Fig. 6A, 6B). Immunoblot analysis demonstrated that Oct4 and Nanog expression were PDGFR inhibitor-IV dose-dependent, with exposure to 0.06 μM inducing enhanced Oct4 and 0.1 μM inducing increased Nanog expression (Fig. 6C). Exposure to 0.06 μM PDGFR inhibitor-IV was also shown to induce a dose-dependent increase in STAT3 (Y705) (Fig. 6C). Thus Oct4, Nanog, and STAT3 (Y705) expression were PDGFR inhibitor-IV dose-dependent.

We then examined the effects of gradually inhibiting ROCK activity. Immunofluorescence analysis showed that ROCK inhibition also affected MSC shape and actin organization (Fig. 6D, 6E). Immunoblot analysis demonstrated that Oct4 expression was H-1152 dose-dependent, with exposure to 2.5 nM inducing enhanced Oct4 expression; however, in contrast to PDGFR inhibitor IV effects, Nanog expression was not increased (Fig. 6F). Exposure to 2.5 nM H-1152 was also shown to produce an increase in STAT3 (Y705) (Fig. 6F). Similar results were also obtained using the ROCK inhibitor Y27632 (data not shown). Thus, while a ROCK-mediated decrease in actomyosin tension produced an increase in Oct4 expression and STAT3 (Y705), it was not sufficient to increase Nanog expression.

Images of low density MSCs exposed to inhibitor H-1152 were analyzed to determine size and shape measurements (Fig. 6G). Compared with untreated controls (Fig. 2A) and PDGFR inhibitor IV treatment (Fig. 2B), MSCs of a similar density exposed to H-1152 adopted an intermediate shape (eccentricity 0.80 ± 0.02; extent 0.40 ± 0.02; form factor 0.17 ± 0.01) (Fig. 6H). However, the nucleus/cytoplasm ratio of H-1152-treated MSCs (0.061 ± 0.005) was similar to control MSCs (0.057 ± 0.005) (Fig. 6I), as was the nuclei shape (data not shown).

Next we determined the effects of gradually inhibiting myosin II ATPase activity. Immunofluorescence analysis showed that following exposure to 5 μM of Blebbistatin, MSCs became more flattened and rounded in shape (Supporting Information Fig. S3A, S3B). Immunoblot analysis demonstrated that exposure to 5 μM of Blebbistatin increased Oct4 and Nanog expression and increased the level of STAT3 (Y705) (Supporting Information Fig. S3C). Thus myosin ATPase II activity may play an important role in regulating Nanog expression.

Finally, we examined the effects of gradually inhibiting actin polymerization. Immunofluorescence analysis showed that, as the dose of Latrunculin B increased, MSC shape became more flattened, with an increasing loss of actin filaments (Supporting Information Fig. S3D, S3E). Immunoblot analysis demonstrated that Oct4 expression was only slightly elevated following exposure to 0.08 μM Latrunculin B, while Nanog expression was variable and STAT3 (Y705) did not increase (Supporting Information Fig. S3F). The results therefore demonstrate that loss of intact actin filaments resulted in uncoordinated expression of Oct4 and Nanog.

Collectively, these results indicate that a decrease in actomyosin tension, leading to a more rounded MSC shape, influences Oct4, Nanog, and STAT3 (Y705) expression.

### PDGFR-Inhibited MSCs Can Differentiate Toward Ectoderm, Endoderm, and Mesoderm Lineages

As a proof of principle, we determined whether PDGFR inhibitor-IV-treated MSCs demonstrated greater multipotency than untreated control MSCs, by differentiating these MSCs toward neural cells or hepatocytes.

**Figure 7:**
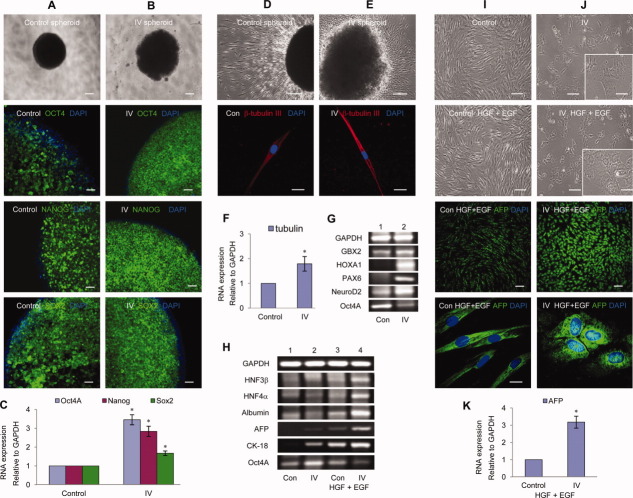
PDGFR inhibition directed differentiation toward neural and hepatocyte lineages. (A, B): Representative bright-field and immunofluorescence images of mesenchymal stem cell (MSC) spheroids after 14 days (A) untreated controls or (B) exposed to 0.1 μM platelet-derived growth factor receptor (PDGFR) inhibitor-IV. Immunofluorescence analysis shows Oct4, Nanog, and Sox2 expression (green) with DAPI-stained nuclei (blue). Scale bars: = 300 μm (bright-field images) and 100 μm (immunofluorescence images). (C): Quantitative RT-PCR analysis of MSC spheroids, untreated controls or exposed to 0.1 μM PDGFR inhibitor-IV for 14 days. Expression of Oct4A, Nanog, and Sox2 relative to GAPDH and normalized to untreated controls. *, *p* < .001 compared with untreated controls, using paired *t* test *n* > 3 separate experiments, error bars represent SD. (D–G): For neural cell differentiation, MSCs were cultured as spheroids in 96-well low cell binding plates for 7 days, using ESC medium with or without 0.1 μM PDGFR inhibitor-IV. Spheroids were cultured for a further 7 days with 0.5 μM retinoic acid, then plated onto 0.1% gelatin, and cultured using NDiff N2B27 neural differentiation medium (Stem Cell Sciences) without PDGFR inhibitor-IV for 7 days. (D, E): Representative bright-field and immunofluorescence images of retinoic acid treated MSC spheroids (D) control, or (E) exposed to PDGFR inhibitor-IV, following 7 days adherent culture using neural cell differentiation medium on a gelatin-coated surface. Immunofluorescence analysis shows β-tubulin III expression (red) with DAPI-stained nuclei (blue). Scale bars = 300 μm (bright-field images) and 20 μm (immunofluorescence images). (F): Quantitative RT-PCR analysis of controls or 0.1 μM PDGFR inhibitor-IV-treated MSC spheroids exposed to retinoic acid for 7 days, expression of β-tubulin III relative to GAPDH and normalized to controls. *, *p* < .001 compared with controls, using paired *t* test *n* > 3 separate experiments; error bars represent SD. (G): RT-PCR analysis of retinoic acid-treated MSC spheroids, [1] controls or [2] exposed to PDGFR inhibitor-IV, expression of neural cell markers; GBX2, HOXA1, PAX6, NeuroD2 and pluripotent marker Oct4A, with GAPDH as a loading control. (H–K): For hepatocyte differentiation, a single step procedure was used. MSCs were cultured in ESC medium with 40 ng/ml HGF and 20 ng/ml EGF (R&D Systems) with or without 0.1 μM PDGFR inhibitor-IV for 14 days. (H): RT-PCR analysis of hepatocyte markers; [1] untreated control, [2] PDGFR inhibitor-IV-treated, [3] control exposed to HGF and EGF, and [4] PDGFR inhibitor-IV-treated exposed to HGF and EGF for 14 days. Expression of HNF3β, HNF4α, Albumin, AFP, Cytokeratin-18 and pluripotent marker Oct4A, with GAPDH as a loading control. (I, J): Representative bright-field and immunofluorescence images of MSC differentiation toward hepatocytes following 14 days culture using serum-free ESC medium (I) untreated controls or exposed to HGF and EGF, (J) exposed to PDGFR inhibitor-IV alone or with HGF and EGF. Immunofluorescence analysis shows AFP expression (green) with DAPI-stained nuclei (blue). Scale bars = 200 μm (bright-field images) and 100 μm (insets show higher magnification images); immunofluorescence images, wide-field = 200 μm; higher magnification = 20 μm. (K): Quantitative RT-PCR analysis of controls or 0.1 μM PDGFR inhibitor-IV-treated MSCs exposed to HGF and EGF for 14 days, expression of AFP relative to GAPDH and normalized to controls. *, *p* < .001 compared with controls, using paired *t*-test *n* > 3 separate experiments; error bars represent SD. Abbreviations: AFP, α-fetoprotein; DAPI, 4′,6-diamino-2-phenylindole; EGF, epidermal growth factor; GAPDH, glyceraldehyde 3-phosphate dehydrogenase; HGF, hepatocyte growth factor; HNF, hepatocyte nuclear factor.

For neural cell differentiation, MSCs were cultured as spheroids and exposed to retinoic acid [37]. Immunofluorescence analysis revealed that, compared with control MSC spheroids (Fig. 7A), PDGFR inhibitor-IV-treated spheroids expressed widespread and abundant Oct4, Nanog, and Sox2 (Fig. 7B). Quantitative RT-PCR demonstrated that compared with control MSC spheroids, PDGFR inhibitor-IV treatment increased Oct4A (3.5 ± 0.3-fold), Nanog (2.8 ± 0.3-fold), and Sox2 (1.7 ± 0.2-fold) (Fig. 7C). When these spheroids were exposed to neural cell differentiation conditions, they quickly developed elongated spindle-shaped outgrowths, which were positive for β-tubulin III (Fig. 7D, 7E). Quantitative RT-PCR demonstrated that compared with control MSC spheroids, PDGFR inhibitor-IV treatment increased β-tubulin III expression (1.8 ± 0.3-fold) (Fig. 7F). In addition, RT-PCR demonstrated PDGFR inhibitor-IV-treated MSC spheroids upregulated expression of neuron markers GBX2 and NeuroD2 and induced HOXA1 and PAX6 expression, while OctA was markedly decreased (Fig. 7G). Therefore, PDGFR inhibitor-IV-treated MSC spheroids displayed greater potential to differentiate toward neural cells.

We next examined whether PDGFR inhibitor-IV-treated MSCs could be differentiated toward hepatocytes. In this analysis, we used a single-step exposure of MSCs to hepatocyte growth factor (HGF) and EGF [38]. RT-PCR analysis of control MSCs demonstrated no marked expression of hepatocyte transcripts (Fig. 7H; lane 1); however, PDGFR inhibitor-IV MSCs expressed cytokeratin-18 and α-Fetoprotein (AFP) (Fig. 7H; lane 2). While control MSCs exposed to HGF/EGF expressed albumin, AFP, and cytokeratin-18 transcripts (Fig. 7H; lane 3), PDGFR inhibitor-IV MSCs exposed to HGF/EGF showed marked upregulation in HNF3β, HNF4α, albumin, AFP, and cytokeratin-18 expression, and Oct4 was notably suppressed (Fig. 7H; lane 4). Compared with control MSCs, exposure to HGF/EGF produced no detectable change in MSC shape (Fig. 7I) but induced PDGFR inhibitor-IV MSCs to became more rounded (Fig. 7J). While HGF/EGF stimulated control and PDGFR inhibitor-IV-treated MSCs were positive for AFP (Fig. 7I, 7J), quantitative RT-PCR demonstrated that PDGFR inhibitor-IV treatment increased AFP expression (3.2 ± 0.3-fold) (Fig. 7K). Thus PDGFR inhibitor-IV MSCs exhibited enhanced capacity for hepatocyte differentiation.

It is well-established that MSCs can be readily induced to differentiate to adipocytes, osteocytes, or chondrocytes [1, 3]. However, we wished to determine whether, compared with control MSCs, exposure to PDGFR inhibitor-IV modulated their ability to differentiate toward mesoderm lineages. Following differentiation, immunofluorescence analysis demonstrated that both control and PDGFR inhibitor-IV-treated MSCs expressed markers for chondrocytes (aggrecan) (Supporting Information Fig. S4A), adipocytes (Bodipy and fatty acid-binding protein (FABP)-4) (Supporting Information Fig. S4B), and osteocytes (osteocalcin) (Supporting Information Fig. S4C). Quantitative RT-PCR was also used to determine the expression of two additional markers for chondrocyte, adipocyte, or osteocyte differentiation. Compared with control MSCs, PDGFR inhibitor-IV treatment significantly increased the expression of the chondrocyte marker collagen type IX and the adipocyte markers adipocyte protein-2 (AP-2) and peroxisome proliferator-activated receptor 2 (PPAR2) (Supporting Information Fig. S4D).

Taken together, these results demonstrate that MSCs pretreated with PDGFR inhibitor-IV can be induced to differentiate toward all three germline lineages, demonstrating their increased multipotency. This study therefore identifies inhibition of PDGFR signaling as a primary regulator of Oct4 and Nanog expression and of MSC potency.

## DISCUSSION

To enhance therapeutic applications, we investigated whether MSC multipotency could be increased by regulating signaling through their abundant PDGFRs, which are key regulators of cell differentiation and mesenchymal tissue formation. Blocking PDGFRs and downstream cAbl signaling with a small molecular inhibitor upregulated Oct4 and Nanog in mechanisms involving JAK-STAT3, MEK and EGFR signaling, and actomyosin contractility. These mechanisms induced a more rounded MSC shape and increased MSC multipotency.

PDGFR inhibitor-IV induced not only a more rounded MSC shape but also a significant change in nuclear shape and size. Changes to the actin cytoskeleton, which is interlinked to the nuclear envelope, can modulate nuclear mechanotransduction resulting in nuclear shape changes, chromatin reorganization, and regulation of gene transcription [39]. Furthermore, ESC nuclei, which are characteristically large and round, have been shown to change their shape and stiffness as the cells differentiate [40]. While little is known about the relationship between cell shape and gene expression, PDGFR inhibitor-IV-induced MSC nuclei shape change is likely to play an important role in regulating nuclear Oct4 and Nanog expression and STAT3 (Y705) translocation.

Changes in actin cytoskeletal organization, which influences cell shape, were also found to be an important regulator of MSC potency, since decreasing ROCK or myosin II activity induced a more rounded MSC shape and increased Oct4 expression. In ESCs, expression of Nanog is regulated by Oct4 and Sox2, which interact and bind to the *Nanog* promoter to increase its activity [41]. Thus our demonstration that decreased actomyosin contractility can upregulate Oct4 identifies a novel mechanism which may regulate stem cell potency.

Actomyosin contractility is regulated by a balance between the levels of RhoA-ROCK and Rac1 activity, which, respectively, increase or decrease actin stress fiber assembly [32]. Stimulation of PDGFRα or PDGFRβ has been shown to activate RhoA and its downstream effector ROCK [8, 42], which increases myosin light chain phosphorylation and actomyosin contractility. Therefore, inhibition of either PDGFRα or PDGFRβ signaling would be expected to reduce actomyosin tension. In this study, PDGFRβ knockdown was shown to increase Oct4A and Nanog more than PDGFRα knockdown but neither individual knockdown affected cell shape. In comparison, exposure to PDGFR inhibitor-IV increased Oct4A and Nanog more than the knockdown of either PDGFRα or PDGFRβ and also induced more rounded MSC shape. Thus, while individual knockdowns demonstrate that distinct PDGFR signaling can regulate Oct4 and Nanog expression, a combination of PDGFR and cAbl inhibition is required for cell shape change and increased MSC potency.

PDGF-induced activation of cytoplasmic cAbl plays an important role in mediating actin assembly and regulation of cell shape [13]. In neurons, inhibition of cAbl signaling can reduce RhoA-ROCK activity and actomyosin contraction [43]. However, the resulting effect is dependent on the cellular context; therefore, the outcome will likely be determined by the balance between Rac and Rho and the effects of other signaling molecules regulated by the Rho-ROCK pathway. In this study, we demonstrated cAbl (Y412) in nuclear extracts, which suppressed PDGFR inhibition. Thus inhibiting cAbl signaling may not only increase Oct4 expression indirectly by decreasing actomyosin tension but may also regulate cellular differentiation due to reduced nuclear cAbl activity.

While actomyosin tension, mediated by ROCK or myosin II activity, has been shown to be pivotal in specifying MSC lineage commitment [11, 44], our results demonstrate that PDGFR inhibition is also crucial for enhancing MSC multipotency. Compared with control MSC spheroids, those exposed to PDGFR inhibitor-IV markedly upregulated Oct4, Nanog, and Sox2 and could be induced to express neuronal markers. Thus, inhibition of PDGFRs and cAbl signaling drives dedifferentiation and increases multipotency.

Our results indicated that actomyosin contractility that directs MSC shape may control STAT3 (Y705) nuclear translocation. There is now increasing evidence that the Rho family of small GTPases may regulate STAT3 nuclear translocation [33], and the mechanisms involved are beginning to be defined. Recent reports suggest that activated Rac1 and STAT3 (Y705) form a complex with MgcRacGAP, which acts as chaperone for nuclear translocation [45]. EGFR signaling is known to activate Rac1 [46], which also regulates actomyosin contractility and cell shape, thus EGFR-activated Rac1 may not only support PDGFR inhibitor-IV induced cell rounding but also STAT3 nuclear import. Since JAK-STAT3 signaling was required for the PDGFR inhibitor-IV-induced rounded MSC shape, and inhibition of JAK activity partly restored an elongated shape, JAK-STAT3 regulation of Rac1 activity [34] may modulate actomyosin tension and STAT3 nuclear translocation.

We demonstrated that MEK signaling was also essential for the PDGFR inhibitor-IV-induced rounded MSC shape, since inhibition of MEK activity completely rescued the elongated shape. Active MEK can downregulate ROCK activity, decreasing actin stress fiber assembly and actomyosin contractility, whereas MEK inhibition can restore ROCK activity [36]. Our results suggest that MEK signaling may promote a decrease in ROCK activity and actomyosin tension, thereby facilitating the PDGFR inhibitor-IV-induced rounded shape. Conversely, inhibiting MEK may restore ROCK activity and actomyosin contractility, which rescues the elongated shape. Inhibition of MEK decreased the level of nuclear Oct4, Nanog, and STAT3 (Y705), further demonstrating that MSC shape and actomyosin contractility regulated STAT3 (Y705) nuclear translocation.

This study has demonstrated that the targeted inhibition of PDGFR signaling increases MSC multipotency. While cell fate is undoubtedly determined by multiple signaling systems working in concert, specifically inhibiting this differentiation pathway provides a novel approach to enhance the potency of MSCs.

## CONCLUSION

By testing the hypothesis that selective inhibition of signaling pathways involved in differentiation may increase stem cell potency, we have shown that PDGFR signaling plays a crucial role in specifying MSC fate and potency. The small molecule PDGFR inhibitor-IV induced MSCs towards a more rounded shape, enhanced expression of Oct4 and Nanog, and supported the expression of definitive endodermal, ectodermal and mesodermal markers. Thus, inhibiting PDGFRs offers a novel approach to modulate the potency of MSCs.

## Notes

Figure S1 PDGFR-IV inhibitor specificity (A-B) ELISA determination of the phosphorylation levels of PDGFRα (Y742) and PDGFRβ (Y1021) relative to corresponding total PDGFRs, expressed by MSCs cultured using ESC medium, when un-stimulated (basal) or stimulated with 25ng/ml PDGF-AA or PDGF-BB for 10 min, following 24hr exposure to (A) 0.1μM PDGFR inhibitor-IV or (B) 20nM PDGFR inhibitor-V. (C) Proteome array analysis to determine the relative phosphorylation levels of 42 different receptor tyrosine kinases (RTKs), expressed by MSCs cultured using ESC medium, when untreated (control) or treated with 0.1μM PDGFR inhibitor-IV for 24 hrs. Each of the array markers has duplicate spots, with PDGFRα and PDGFRβ boxed to highlight their identity, and other prominently phosphorylated RTKs identified as indicated. (D) Immunoprecipitation of cAbl then immunoblot analysis of phospho tyrosine to determine cAbl phosphorylation levels, with total cAbl as loading controls, following 24 hrs exposure to 0.1μM PDGFR inhibitor-IV, 20nM PDGFR inhibitor-V, or un-treated controls. (E) Immunoblot analysis examining nuclear and cytoplasm expression of phosphorylated cAbl (Y412) with total cAbl and β-actin as loading controls and nuclear membrane marker Lamin B1, following 24 hrs exposure to 0.1μM PDGFR inhibitor-IV or un-treated controls.

Figure S2 PDGFR inhibitor-IV increased pSTAT3 nuclear translocation (A) Immunofluorescence analysis examining nuclear and cytoplasm localized phosphorylated STAT3 (Y705), following 24hr exposure to 0.1μM PDGFR inhibitor-IV or un-treated controls. For particle analysis, images were processed using ImageJ software and particle analysis plugin. Similar best-fit lower threshold values were determined to reduce signal background of the corresponding green channel, the pixel size range was set at a minimum of 1 and maximum of 1000. Representative images show STAT3 (Y705) (green) with DAPI stained nuclei (blue), and green channel images only, having the same threshold value and particle size range. Scale bars: 20μm. (B) STAT3 (Y705) nuclear / cytoplasm ratios, following 24hr exposure to 0.1μM PDGFR inhibitor-IV or un-treated controls. Mean number of particles localized in the nucleus or cytoplasm, derived from green channel images of ten different single cells, having the same threshold value and particle size range. Scale bars: 20μm. *P < 0.001 compared to un-treated controls, using paired t-test, error bars represent s.d. (C) Quantitative RT-PCR analysis examining the expression of Oct4A and Nanog relative to GAPDH and normalized to un-treated controls, following 1hr exposure to 50ng/ml EGF. (D) Immunoblot analysis of phosphorylated STAT3 (Y705) with total STAT3 as loading controls, following 24 hrs exposure to 20nM JAK inhibitor or un-treated controls. (E) Immunoblot analysis of phosphorylated ERK1/2 with β-actin as loading controls, following 24 hrs exposure to 20μM MEK inhibitor or un-treated controls.

Figure S3 Blebbistatin reduced actomyosin tension, increased Oct4, Nanog and STAT3 (Y705) (A,D) Immunofluorescence analysis examining F-actin filament organization and MSC shape, following 24hr exposure to an increasing dose of (A) Blebbistatin (0-5μM) or (D) Latrunculin B (0-0.2μM). Representative images show F-actin filaments (red) and nuclei (blue). Scale bars: 20μm. (B,E) Representative bright-field images of MSC morphology following 24 hrs exposure to an increasing dose of (B) Blebbistatin (0-5μM) or (E) Latrunculin B (0-0.2μM). Scale bars: 100μm. (C,F) Immunoblot analysis determining Oct4, Nanog and phosphorylated STAT3 (Y705) expression levels, with β-actin and total STAT3 as corresponding loading controls, following 24hr exposure to an increasing dose of (C) Blebbistatin (0-5μM) or (F) Latrunculin B (0- 0.2μM).

Figure S4 PDGFR inhibited MSCs differentiated towards chondrocytes, adipocytes and osteocytes (A-D) For adipocyte, osteocyte and chondrocyte differentiation, MSCs were pre-cultured in ESC medium with or without 0.1μM PDGFR inhibitor-IV for 3 days, then differentiated using defined adipocyte, osteocyte or chondrocyte differentiation medium (R&D Systems; MSC functional identification kit SC006), with or without PDGFR inhibitor-IV for 7 days. (A) Immunofluorescence analysis examining MSCs exposed to chondrocyte differentiation medium (i) un-differentiated control (ii) differentiated control (iii) PDGFR inhibitor-IV. Representative images show aggrecan (green), F-actin filaments (red) with nuclei (blue). (B) Immunofluorescence analysis examining MSCs exposed to adipocyte differentiation medium (i) un-differentiated control (ii) differentiated control (iii) PDGFR inhibitor-IV. Representative images show bodipy (green), FABP-4 (red) with nuclei (blue). (C) Immunofluorescence analysis examining MSCs exposed to osteocyte differentiation medium (i) un-differentiated control (ii) differentiated control (iii) PDGFR inhibitor-IV. Representative images show osteocalcin (green), F-actin filaments (red) with nuclei (blue). Scale bars: 20μm. (D) Quantitative RT-PCR analysis examining expression of chondrocyte markers collagen type II (Col-II), collagen type IX (Col-IX), adipocyte markers AP-2, PPAR2α and osteocyte markers osteopontin (OP), alkaline phosphatase (AP) relative to GAPDH, following 7 days culture using defined differentiation medium in the presence or absence (control) of 0.1μM PDGFR inhibitor-IV. *P < 0.001 compared to un-treated controls, using paired t-test n > 3 separate experiments, error bars represent s.d.
