# Supplementary figures and images for: Inhibition of Platelet-Derived Growth Factor Receptor Signaling Regulates Oct4 and Nanog Expression, Cell Shape, and Mesenchymal Stem Cell Potency
Source: Stem Cells. 2012 Feb 14;30(3):548–60. doi: 10.1002/stem.1015 (PMC3537888; doi:10.1002/stem.1015)

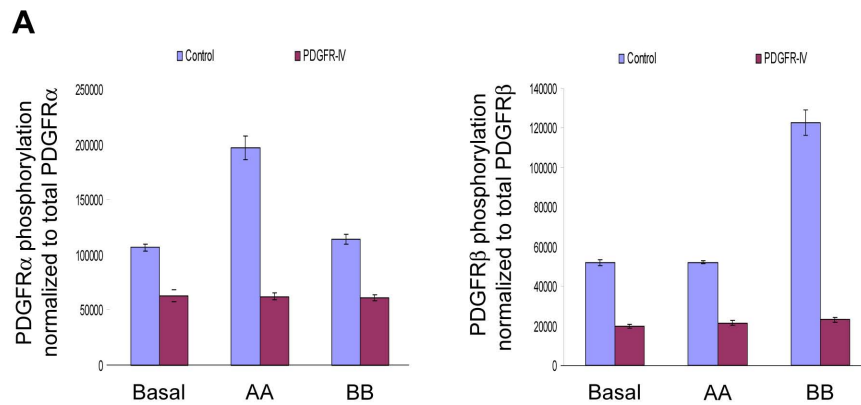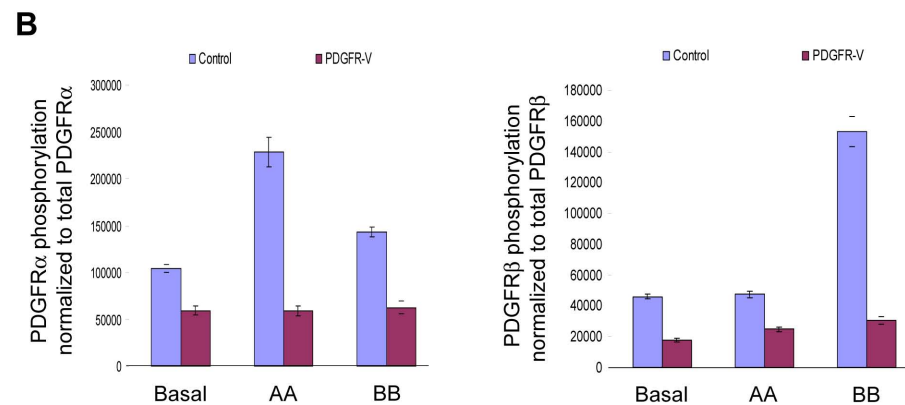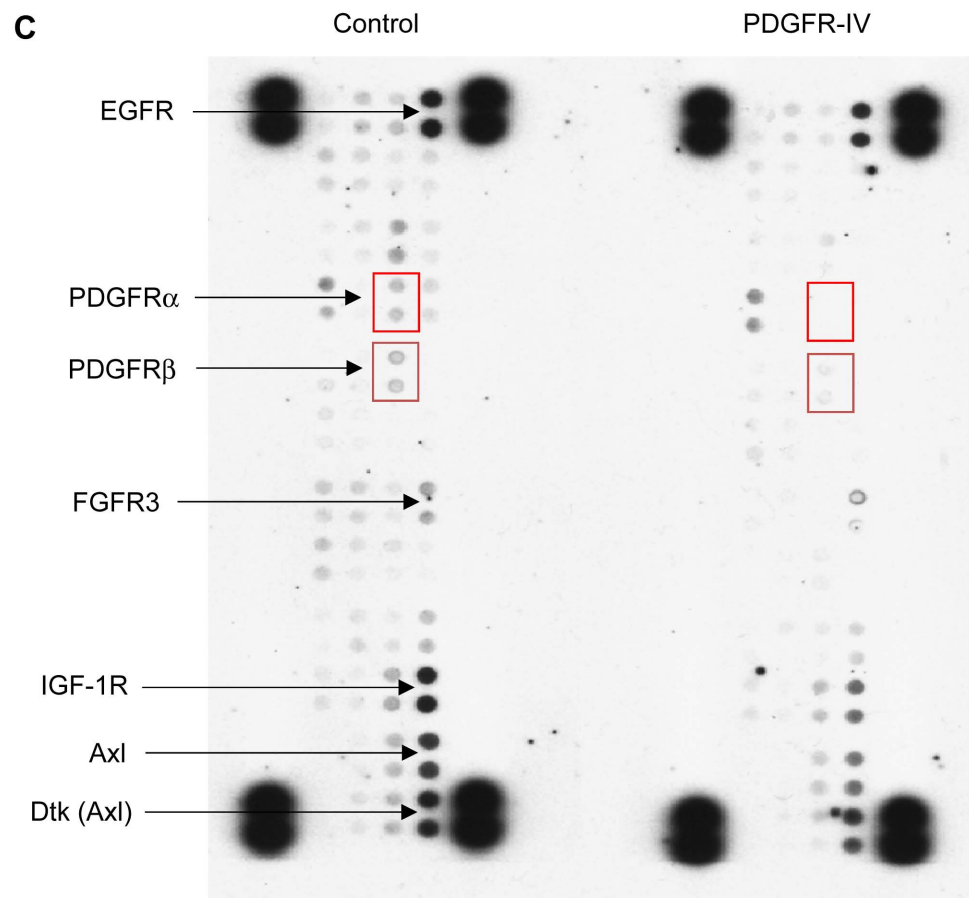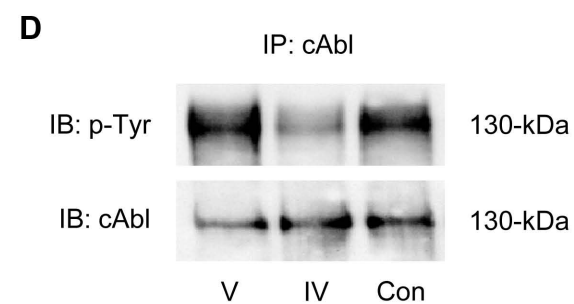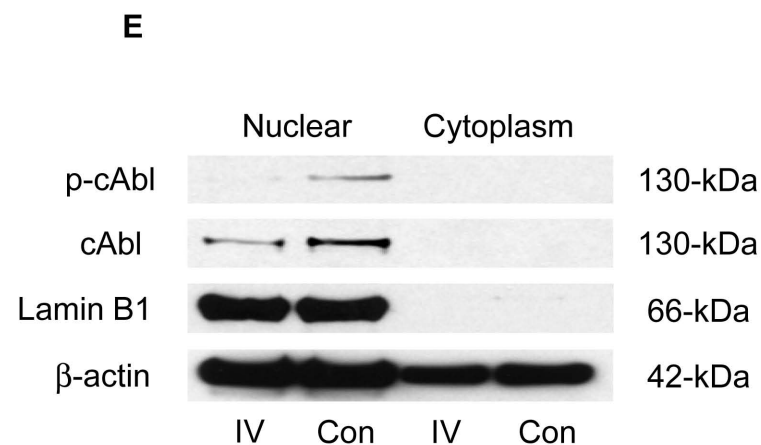

Supplement: Figure S1 — PDGFR-IV inhibitor specificity (A-B) ELISA determination of the phosphorylation levels of PDGFRα (Y742) and PDGFRβ (Y1021) relative to corresponding total PDGFRs, expressed by MSCs cultured using ESC medium, when un-stimulated (basal) or stimulated with 25ng/ml PDGF-AA or PDGF-BB for 10 min, following 24hr exposure to (A) 0.1μM PDGFR inhibitor-IV or (B) 20nM PDGFR inhibitor-V. (C) Proteome array analysis to determine the relative phosphorylation levels of 42 different receptor tyrosine kinases (RTKs), expressed by MSCs cultured using ESC medium, when untreated (control) or treated with 0.1μM PDGFR inhibitor-IV for 24 hrs. Each of the array markers has duplicate spots, with PDGFRα and PDGFRβ boxed to highlight their identity, and other prominently phosphorylated RTKs identified as indicated. (D) Immunoprecipitation of cAbl then immunoblot analysis of phospho tyrosine to determine cAbl phosphorylation levels, with total cAbl as loading controls, following 24 hrs exposure to 0.1μM PDGFR inhibitor-IV, 20nM PDGFR inhibitor-V, or un-treated controls. (E) Immunoblot analysis examining nuclear and cytoplasm expression of phosphorylated cAbl (Y412) with total cAbl and β-actin as loading controls and nuclear membrane marker Lamin B1, following 24 hrs exposure to 0.1μM PDGFR inhibitor-IV or un-treated controls. [file stem0030-0548-SD1.pdf]

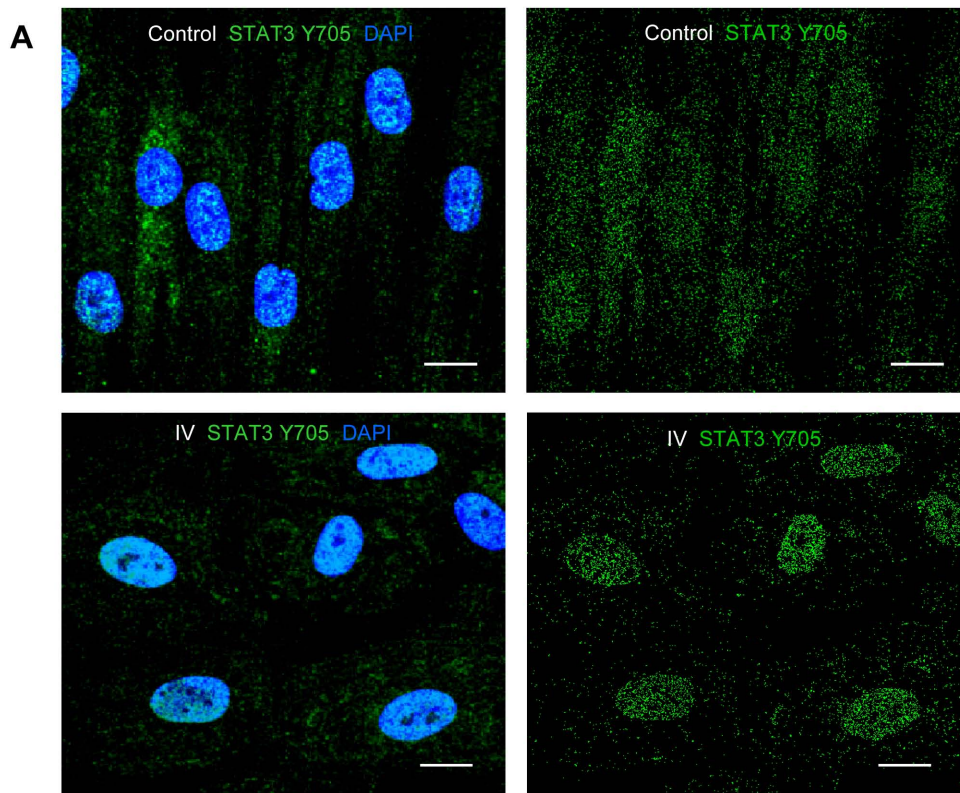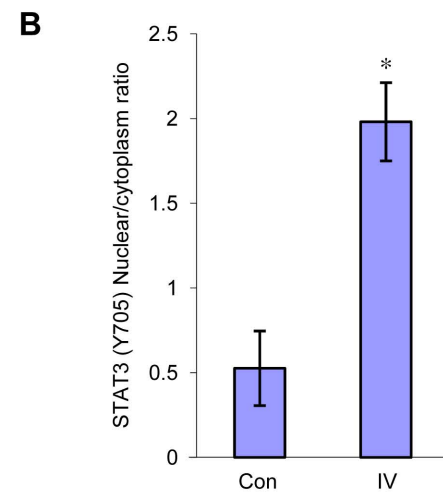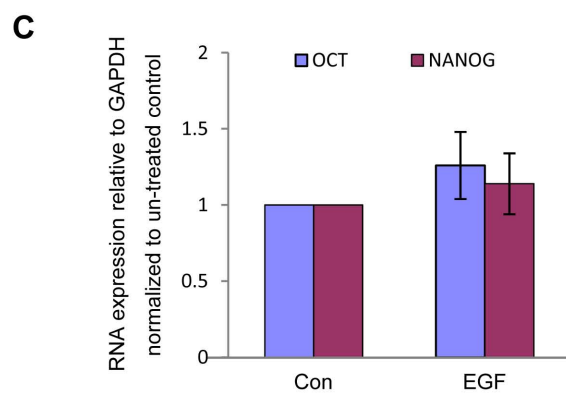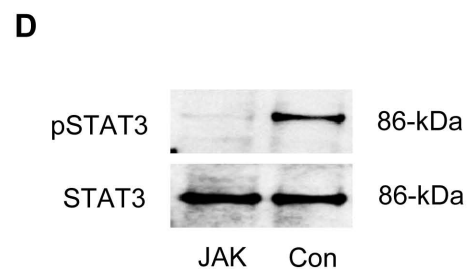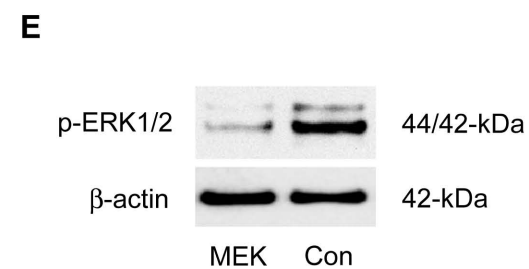

Supplement: Figure S2 — PDGFR inhibitor-IV increased pSTAT3 nuclear translocation (A) Immunofluorescence analysis examining nuclear and cytoplasm localized phosphorylated STAT3 (Y705), following 24hr exposure to 0.1μM PDGFR inhibitor-IV or un-treated controls. For particle analysis, images were processed using ImageJ software and particle analysis plugin. Similar best-fit lower threshold values were determined to reduce signal background of the corresponding green channel, the pixel size range was set at a minimum of 1 and maximum of 1000. Representative images show STAT3 (Y705) (green) with DAPI stained nuclei (blue), and green channel images only, having the same threshold value and particle size range. Scale bars: 20μm. (B) STAT3 (Y705) nuclear / cytoplasm ratios, following 24hr exposure to 0.1μM PDGFR inhibitor-IV or un-treated controls. Mean number of particles localized in the nucleus or cytoplasm, derived from green channel images of ten different single cells, having the same threshold value and particle size range. Scale bars: 20μm. *P < 0.001 compared to un-treated controls, using paired t-test, error bars represent s.d. (C) Quantitative RT-PCR analysis examining the expression of Oct4A and Nanog relative to GAPDH and normalized to un-treated controls, following 1hr exposure to 50ng/ml EGF. (D) Immunoblot analysis of phosphorylated STAT3 (Y705) with total STAT3 as loading controls, following 24 hrs exposure to 20nM JAK inhibitor or un-treated controls. (E) Immunoblot analysis of phosphorylated ERK1/2 with β-actin as loading controls, following 24 hrs exposure to 20μM MEK inhibitor or un-treated controls. [file stem0030-0548-SD2.pdf]

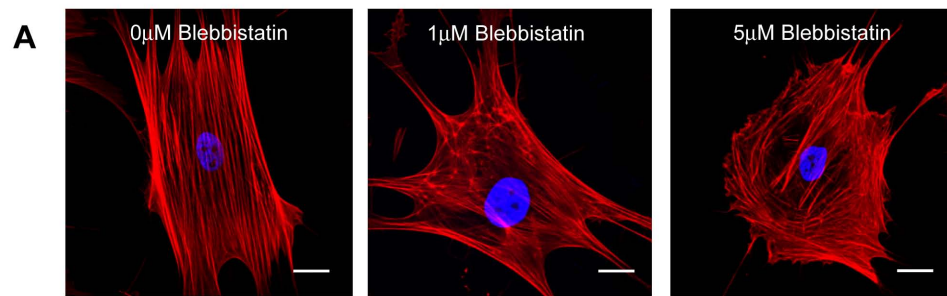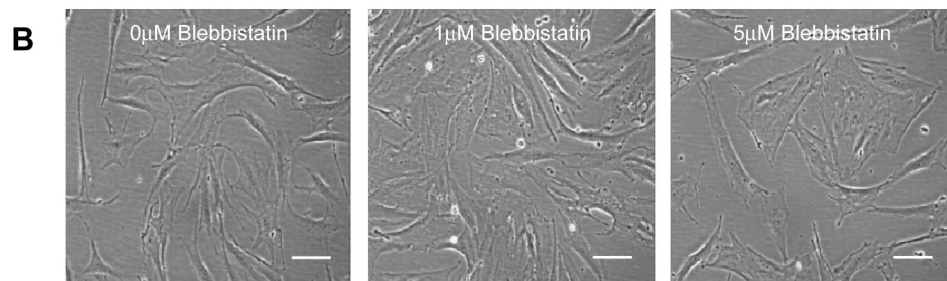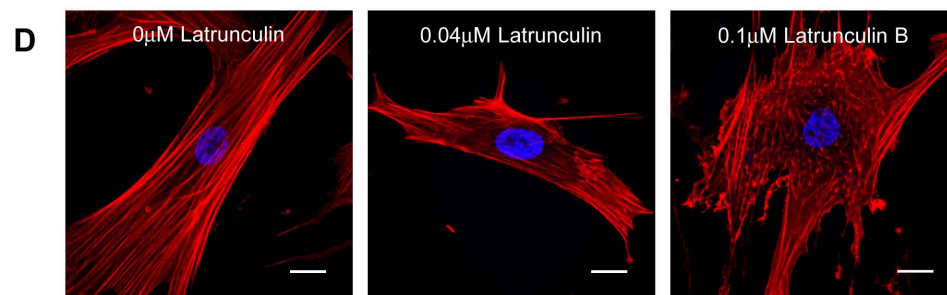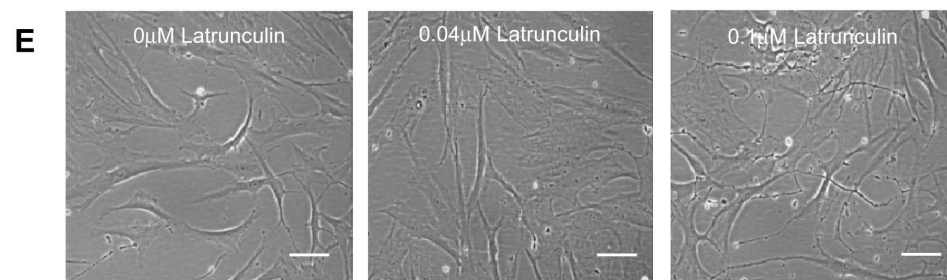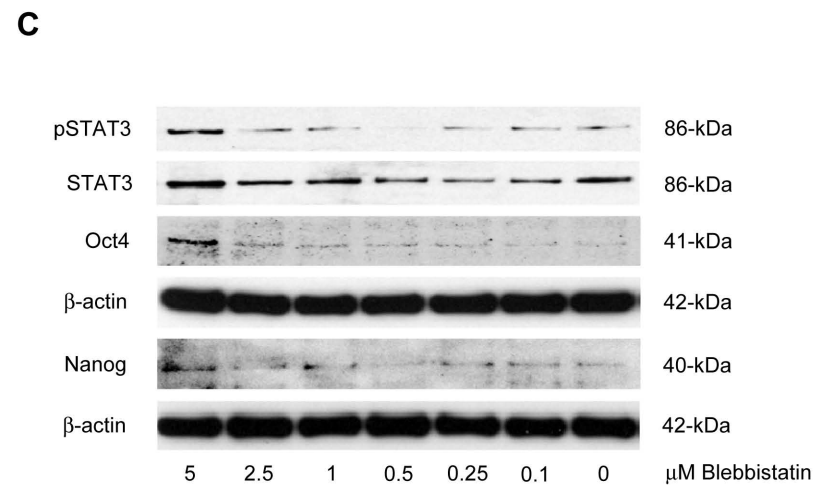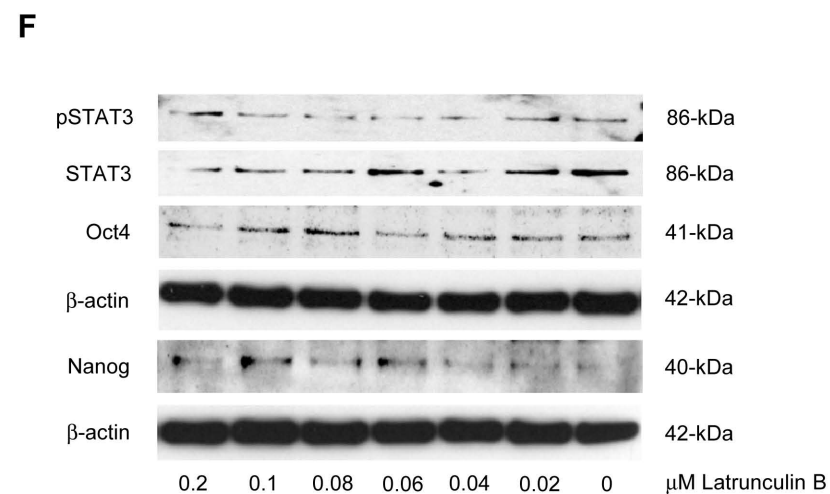

Supplement: Figure S3 — Blebbistatin reduced actomyosin tension, increased Oct4, Nanog and STAT3 (Y705) (A,D) Immunofluorescence analysis examining F-actin filament organization and MSC shape, following 24hr exposure to an increasing dose of (A) Blebbistatin (0-5μM) or (D) Latrunculin B (0-0.2μM). Representative images show F-actin filaments (red) and nuclei (blue). Scale bars: 20μm. (B,E) Representative bright-field images of MSC morphology following 24 hrs exposure to an increasing dose of (B) Blebbistatin (0-5μM) or (E) Latrunculin B (0-0.2μM). Scale bars: 100μm. (C,F) Immunoblot analysis determining Oct4, Nanog and phosphorylated STAT3 (Y705) expression levels, with β-actin and total STAT3 as corresponding loading controls, following 24hr exposure to an increasing dose of (C) Blebbistatin (0-5μM) or (F) Latrunculin B (0- 0.2μM). [file stem0030-0548-SD3.pdf]

**A**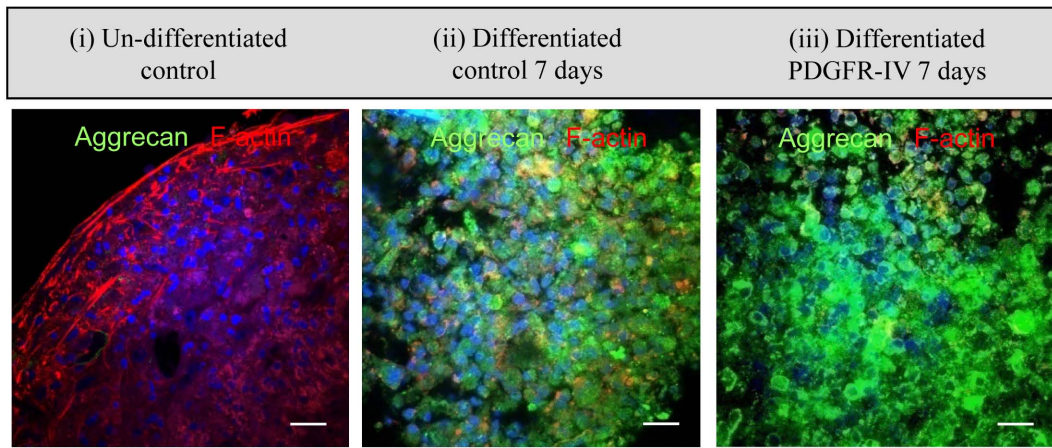**B**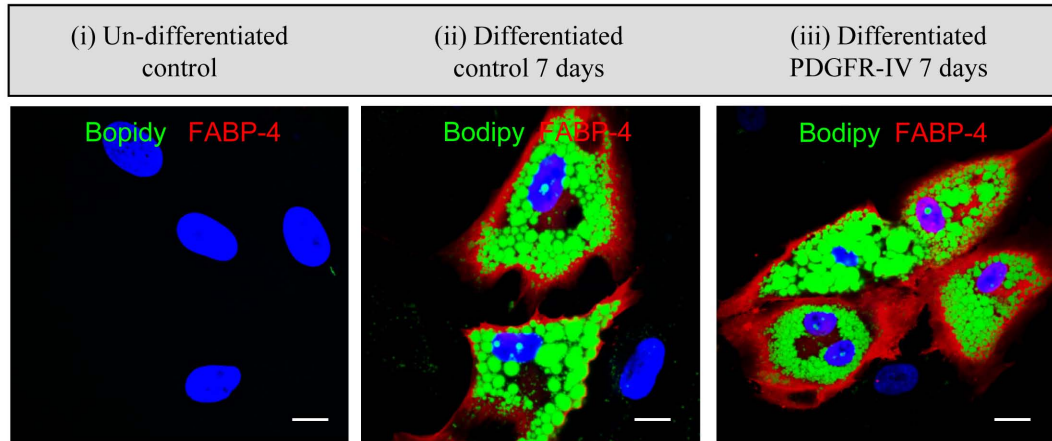**C**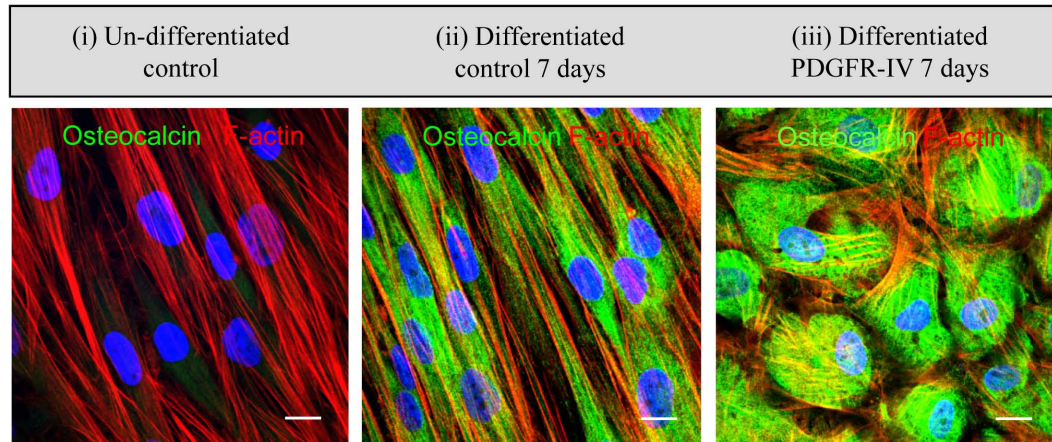**D**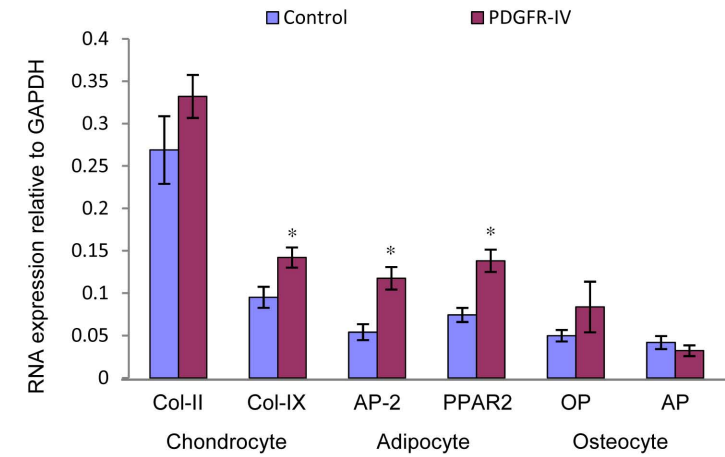

Supplement: Figure S4 — PDGFR inhibited MSCs differentiated towards chondrocytes, adipocytes and osteocytes (A-D) For adipocyte, osteocyte and chondrocyte differentiation, MSCs were pre-cultured in ESC medium with or without 0.1μM PDGFR inhibitor-IV for 3 days, then differentiated using defined adipocyte, osteocyte or chondrocyte differentiation medium (R&D Systems; MSC functional identification kit SC006), with or without PDGFR inhibitor-IV for 7 days. (A) Immunofluorescence analysis examining MSCs exposed to chondrocyte differentiation medium (i) un-differentiated control (ii) differentiated control (iii) PDGFR inhibitor-IV. Representative images show aggrecan (green), F-actin filaments (red) with nuclei (blue). (B) Immunofluorescence analysis examining MSCs exposed to adipocyte differentiation medium (i) un-differentiated control (ii) differentiated control (iii) PDGFR inhibitor-IV. Representative images show bodipy (green), FABP-4 (red) with nuclei (blue). (C) Immunofluorescence analysis examining MSCs exposed to osteocyte differentiation medium (i) un-differentiated control (ii) differentiated control (iii) PDGFR inhibitor-IV. Representative images show osteocalcin (green), F-actin filaments (red) with nuclei (blue). Scale bars: 20μm. (D) Quantitative RT-PCR analysis examining expression of chondrocyte markers collagen type II (Col-II), collagen type IX (Col-IX), adipocyte markers AP-2, PPAR2α and osteocyte markers osteopontin (OP), alkaline phosphatase (AP) relative to GAPDH, following 7 days culture using defined differentiation medium in the presence or absence (control) of 0.1μM PDGFR inhibitor-IV. *P < 0.001 compared to un-treated controls, using paired t-test n > 3 separate experiments, error bars represent s.d. [file stem0030-0548-SD4.pdf]
