# Supplementary material for: Inhibition of Platelet-Derived Growth Factor Receptor Signaling Regulates Oct4 and Nanog Expression, Cell Shape, and Mesenchymal Stem Cell Potency
Source: Stem Cells. 2012 Feb 14;30(3):548–60. doi: 10.1002/stem.1015 (PMC3537888; doi:10.1002/stem.1015)
Supplement: Supplementary file 5 [file stem0030-0548-SD5.pdf]

**Table S1 Details of the inhibitors.**

| Inhibitor | Specificity |
|---|---|
| PDGFR inhibitor-IV | PDGFRα ($IC_{50}$ = 45nM), PDGFRβ ($IC_{50}$ = 4.2nM) and c-Abl ($IC_{50}$ = 22nM). Exhibits less activity towards Lck ($IC_{50}$ = 100nM), c-Src ($IC_{50}$ = 185nM) and Fyn ($IC_{50}$ = 378nM). Exhibits little or no inhibition towards VEGFR ($IC_{50}$ = 3.1μM), bFGFR-1 ($IC_{50}$ = 45.8μM), or EGFR ($IC_{50}$ > 100μM). [20] |
| PDGFR inhibitor-V | PDGFRα ($IC_{50}$ < 10nM), PDGFRβ ($IC_{50}$ < 10nM) and c-kit ($IC_{50}$ = 87nM). Exhibits little or no activity towards c-Abl ($IC_{50}$ > 1μM), FGFR-2 ($IC_{50}$ > 1μM), or EGFR ($IC_{50}$ > 1μM). [23] |
| EGFR (PD168393) | EGFR ($IC_{50}$ = 700pM). Does not inhibit other protein kinases. |
| FGFR (341608) | FGFR-1 ($IC_{50}$ < 60nM). Exhibits less activity towards PDGFRβ , EGFR, insulin receptor or c-Src ($IC_{50}$ > 50μM). |
| MEK (PD98059) | PDGF stimulated MEK ($IC_{50}$ ~ 10μM). No significant effect on protein kinase C, cAMP-dependent kinase, PI3K, Raf kinase, insulin receptor, EGFR or PDGFR. |
| PI3K (LY294002) | PI3K ($IC_{50}$ = 1.4μM). Exhibits no activity towards MAPK, protein kinase C, c-Src or EGFR at 50μM. |
| STAT3 (Inhibitor VI) | Inhibits DNA-binding activity; STAT3 ($IC_{50}$ = 86±33μM) STAT1 ($IC_{50}$ > 300μM). Inhibits STAT3 phosphorylation, with no significant effect on ERK1/2 or Src phosphorylation. |
| GSK-3 (Inhibitor IX) | GSK-3α/β ($IC_{50}$ = 5nM). Exhibits less activity towards Cdk5/p25 ($IC_{50}$ = 83nM), Cdk2/cyclin A ($IC_{50}$ = 300M). Exhibits little or no inhibition towards MAPKs, protein kinase A, or protein kinase C ($IC_{50}$ ≥ 10μM). |
| JAK (Inhibitor I) | JAK1 ($IC_{50}$ = 15nM for murine JAK1), JAK2 ($IC_{50}$ = 1nM), JAK3 ($K_i$ = 5nM) and Tyk2 ($IC_{50}$ = 1nM). Inhibits other kinases at much higher concentrations. |

| | |
|---|---|
| Rho-kinase (H-1152) | ROCK ($K_i$ = 1.6nM). Inhibits other kinases only at much higher concentrations. Protein kinase A ($K_i$ = 630nM), protein kinase C ($K_i$ = 9.27μM) and myosin light chain kinase ($K_i$ = 10.1μM). |
| Blebbistatin (203391) | Inhibits ATPase activity ($IC_{50}$ ~ 2μM) myosin II dependent cellular processes. Inhibits smooth muscle myosin II only at higher concentrations ($IC_{50}$ ~ 80μM). |
| Latrunculin B (428020) | Inhibits actin polymerization and disrupts microfilament organization. 10- to 100-fold more potent than cytochalasins. |

All the inhibitors were obtained from Merck.
