# Supplementary material for: Inhibition of Platelet-Derived Growth Factor Receptor Signaling Regulates Oct4 and Nanog Expression, Cell Shape, and Mesenchymal Stem Cell Potency
Source: Stem Cells. 2012 Feb 14;30(3):548–60. doi: 10.1002/stem.1015 (PMC3537888; doi:10.1002/stem.1015)
Supplement: Supplementary file 6 [file stem0030-0548-SD6.pdf]

Table S2 PCR primer sequences.

Gene (product size)	Forward primer	Reverse primer
GAPDH (71-bp)	5'-AAGGGCATCCTGGGCTAC-3'	5'-GTGGAGGAGTGGGTGTCG-3'
Oct4A (83-bp)	5'-CCCTCCAGGTGGTGGAGG-3'	5'-GGCCTTGGAAGCTTAGCC-3'
Oct4B (70-bp)	5'-AGCGATCAAGCAGCGACT-3'	5'-GGTCCCCCTGAGAAAGGA-3'
Nanog (70-bp)	5'-CCTCCTCCCATCCCTCAT-3'	5'-GGATGGGCATCATGGAAA-3'
GBX2 (77-bp)	5'-CTCGCTGGAGAGCGATGT-3'	5'-CCGGGTCTTCCTCCTTGT-3'
HOXA1 (76-bp)	5'-CGCTCCCGCTGTTTACTC-3'	5'-CCCAGCATAACCCTGGTG-3'
PAX6 (81-bp)	5'-TGTCCGAGGGGGTCTGTA-3'	5'-TTTCGCTAGCCAGGTTGC-3'
NeuroD2 (99-bp)	5'-GACACCCCCATCCTACCC-3'	5'-ACAGGCCACCCACAGGTA-3'
β-tubulin III (91-bp)	5'-CGAGGCGCTCTACGACAT-3'	5'-GCTCATGGTGGCCGATAC-3'
PEPCK (93-bp)	5'-CTTTGGCAGTGGGTACGG-3'	5'-AGCCACCCTTCCTCCTTG-3'
HNF3β (85-bp)	5'-ACCACCCGTTCTCCATCA-3'	5'-TCCATTTTGTGGGGTTGG-3'
HNF4α (80-bp)	5'-CTCCATCAATGCGCTCCT-3'	5'-GAATGTCGCCGTTGATCC-3'
TAT (75-bp)	5'-CGATGTCCCCATCCTGTC-3'	5'-AGGATCCAGCCCAACCTC-3'
Albumin (93-bp)	5'-AAGGCTTCGTCTGCCAAA-3'	5'-GCTCAGGCGAGCTACTGC-3'
α-Fetoprotein (95-bp)	5'-ACCTCGTCGGAGCTGATG-3'	5'-TCGCCACAGGCCAATAGT-3'
Cytokeratin-18 (96-bp)	5'-CGACGCTCACAGAGCTGA-3'	5'-CCCTCAGGCTGTTCTCCA-3'
Collagen type IIA1 86-bp)	5'-GGCTCCCAGAACATCACC-3'	5'-ATGAGCAGGGCCTTCTTG-3'
Collagen type IXA2 (90-bp)	5'AGGAATAGGGCGGCTTTC3'	5'-AAGATGGCCAGTGGAGGA-3'
AP-2 (94-bp)	5'-GCCAACGTTACCCTGCTC-3'	5'-TGGCAGGAAATTCGGTTT-3'
PPAR2γ (91-bp)	5'-CTGCGAAAGCCTTTTGGT-3'	5'-TTGCCAAGTCGCTGTCAT-3'
Osteopontin (70-bp)	5'-TCCAAAGTCAGCCGTGAA-3'	5'-TGGGGTCTACAACCAGCA-3'

Alkaline Phosphatase (84-bp)	5’-CTTGGGCAGGCAGAGAGT-3’	5’-GCCTCTGGGTCTGGAGAA-3’

To discriminate between parental Oct4 transcripts and pseudogenes, the Oct4A forward primer sequence incorporated a unique polymorphism located in the gene at position 48 [25].
