# Supplementary material for: Inhibition of Platelet-Derived Growth Factor Receptor Signaling Regulates Oct4 and Nanog Expression, Cell Shape, and Mesenchymal Stem Cell Potency
Source: Stem Cells. 2012 Feb 14;30(3):548–60. doi: 10.1002/stem.1015 (PMC3537888; doi:10.1002/stem.1015)
Supplement: Supplementary file 7 [file stem0030-0548-SD7.pdf]

Table S3 Details of the antibodies and stains.

Antibody	Supplier	Application
Oct4 (sc-5279)	Santa Cruz	Immunoblotting (1:100)<br>Immunofluoresence (1:50)
Nanog (AF1997)	R&D Systems	Immunoblotting (1:50)<br>Immunofluoresence (1:50)
Sox2 (AF2018)	R&D Systems	Immunofluoresence (1:50)
SSEA-3 (MAB1434)	R&D Systems	Immunofluoresence (1:50)
SSEA-4 (MAB1435)	R&D Systems	Immunofluoresence (1:50)
PDGFRα (sc-338)	Santa Cruz	Immunoblotting (1:1000)
PDGFRβ (sc-339)	Santa Cruz	Immunoblotting (1:1000)
c-Abl (#2862)	Cell Signaling	Immunoblotting (1:1000)<br>Immunoprecipitation (1:100)
c-Abl (Y412) (#2865S)	Cell Signaling	Immunoblotting (1:1000)
α-Fetoprotein (MAB1368)	R&D Systems	Immunofluoresence (1:50)
β-tubulin III (MAB1637)	Millipore	Immunofluoresence (1:50)
Lamin B1 (ab16048)	Abcam	Immunoblotting (1:2000)
p-STAT3(Y705) (#9131)	Cell Signaling	Immunoblotting (1:1000)<br>Immunofluoresence (1:100)
STAT3 (sc-8019)	Santa Cruz	Immunoblotting (1:1000)
p-ERK1/2 (sc-16982)	Santa Cruz	Immunoblotting (1:1000)
p-Tyr (sc-7020)	Santa Cruz	Immunoblotting (1:1000)
β-actin (A1978)	Sigma-Aldrich	Immunoblotting (1:5000)
Aggrecan (SC006)	R&D Systems	Immunofluoresence (1:200)
FABP-4 (SC006)	R&D Systems	Immunofluoresence (1:200)

Osteocalcin (SC006)	R&D Systems	Immunofluoresence (1:200)
**Stain**	**Supplier**	**Application**
Boron-dipyrromethene (Bodipy) 493/503	Invitrogen	Immunofluoresence staining lipid droplets (1:1000)
Rhodamine-conjugated phalloidin	Invitrogen	Immunofluoresence staining actin filaments (1:1000)
Wheat Germ Agglutinin Alexa Fluor 488 conjugate	Invitrogen	Immunofluoresence staining cell membrane lectins (1:500)
