# Supplementary material for: Inhibition of Platelet-Derived Growth Factor Receptor Signaling Regulates Oct4 and Nanog Expression, Cell Shape, and Mesenchymal Stem Cell Potency
Source: Stem Cells. 2012 Feb 14;30(3):548–60. doi: 10.1002/stem.1015 (PMC3537888; doi:10.1002/stem.1015)
Supplement: Supplementary file 8 [file stem0030-0548-SD8.pdf]

**Table S4  CellProfiler image analysis data.**

**Control**

| | | | | |
|---|---|---|---|---|
| Nuclei | MajorAxisLength | 19.37 | 19.35 | 3.26 |
| Nuclei | MinorAxisLength | 13.63 | 13.46 | 2.47 |
| Nuclei | Orientation | 14.28 | 21.52 | 49.40 |
| Nuclei | Compactness | 1.15 | 1.13 | 0.09 |
| Nuclei | Area | 198.79 | 205.50 | 67.01 |
| Nuclei | Center_X | 274.21 | 263.50 | 116.06 |
| Nuclei | Center_Y | 260.86 | 267.00 | 123.32 |
| Nuclei | Extent | 0.62 | 0.62 | 0.06 |
| Nuclei | Perimeter | 66.66 | 63.87 | 13.79 |
| Nuclei | Solidity | 0.84 | 0.85 | 0.05 |
| Nuclei | FormFactor | 0.57 | 0.57 | 0.13 |
| Nuclei | EulerNumber | 1.00 | 1.00 | 0.00 |
| Cells | Eccentricity | 0.89 | 0.92 | 0.10 |
| Cells | MajorAxisLength | 129.11 | 117.31 | 37.07 |
| Cells | MinorAxisLength | 52.81 | 46.05 | 25.13 |
| Cells | Orientation | 20.65 | 29.24 | 47.73 |
| Cells | Compactness | 2.59 | 2.33 | 1.31 |
| Cells | Area | 3519.39 | 3070.50 | 2020.17 |
| Cells | Center_X | 270.21 | 255.00 | 125.35 |
| Cells | Center_Y | 257.46 | 280.00 | 121.55 |
| Cells | Extent | 0.32 | 0.28 | 0.11 |
| Cells | Perimeter | 656.88 | 568.03 | 256.90 |
| Cells | Solidity | 0.55 | 0.52 | 0.13 |
| Cells | FormFactor | 0.12 | 0.09 | 0.09 |
| Cells | EulerNumber | 0.89 | 1.00 | 0.31 |
| Cytoplasm | Eccentricity | 0.89 | 0.92 | 0.10 |
| Cytoplasm | MajorAxisLength | 131.33 | 119.88 | 36.66 |
| Cytoplasm | MinorAxisLength | 53.56 | 47.19 | 24.94 |
| Cytoplasm | Orientation | 20.62 | 29.05 | 47.69 |
| Cytoplasm | Compactness | 2.84 | 2.46 | 1.52 |
| Cytoplasm | Area | 3391.50 | 2939.00 | 2034.11 |
| Cytoplasm | Center_X | 270.79 | 263.00 | 128.04 |
| Cytoplasm | Center_Y | 259.82 | 277.00 | 122.22 |
| Cytoplasm | Extent | 0.30 | 0.26 | 0.10 |
| Cytoplasm | Perimeter | 709.20 | 626.24 | 253.26 |
| Cytoplasm | Solidity | 0.53 | 0.49 | 0.13 |

## PDGFR inhibitor-IV

| | | | | |
|---|---|---|---|---|
| Nuclei | MajorAxisLength | 18.77 | 18.53 | 3.01 |
| Nuclei | MinorAxisLength | 14.10 | 14.00 | 1.76 |
| Nuclei | Orientation | -6.42 | -0.98 | 50.08 |
| Nuclei | Compactness | 1.06 | 1.05 | 0.07 |
| Nuclei | Area | 205.59 | 201.00 | 52.20 |
| Nuclei | Center_X | 277.63 | 296.00 | 132.04 |
| Nuclei | Center_Y | 253.71 | 248.00 | 142.70 |
| Nuclei | Extent | 0.71 | 0.72 | 0.06 |
| Nuclei | Perimeter | 58.24 | 56.11 | 10.42 |
| Nuclei | Solidity | 0.91 | 0.92 | 0.03 |
| Nuclei | FormFactor | 0.77 | 0.79 | 0.11 |
| Nuclei | EulerNumber | 1.00 | 1.00 | 0.00 |
| Cells | Eccentricity | 0.74 | 0.79 | 0.17 |
| Cells | MajorAxisLength | 66.04 | 62.45 | 21.59 |
| Cells | MinorAxisLength | 39.58 | 38.45 | 11.17 |
| Cells | Orientation | -5.88 | -3.24 | 47.06 |
| Cells | Compactness | 1.43 | 1.40 | 0.28 |
| Cells | Area | 1776.59 | 1423.00 | 1050.11 |
| Cells | Center_X | 277.78 | 298.00 | 134.21 |
| Cells | Center_Y | 252.76 | 250.00 | 143.37 |
| Cells | Extent | 0.48 | 0.46 | 0.09 |
| Cells | Perimeter | 320.43 | 300.60 | 111.69 |
| Cells | Solidity | 0.71 | 0.71 | 0.09 |
| Cells | FormFactor | 0.23 | 0.21 | 0.09 |
| Cells | EulerNumber | 1.00 | 1.00 | 0.00 |
| Cytoplasm | Eccentricity | 0.74 | 0.79 | 0.17 |
| Cytoplasm | MajorAxisLength | 68.08 | 63.81 | 21.81 |
| Cytoplasm | MinorAxisLength | 40.71 | 39.60 | 11.15 |
| Cytoplasm | Orientation | -7.98 | -4.82 | 47.89 |
| Cytoplasm | Compactness | 1.69 | 1.62 | 0.37 |
| Cytoplasm | Area | 1635.78 | 1337.00 | 1029.93 |
| Cytoplasm | Center_X | 275.27 | 298.00 | 137.89 |
| Cytoplasm | Center_Y | 254.53 | 258.00 | 141.79 |
| Cytoplasm | Extent | 0.43 | 0.41 | 0.08 |
| Cytoplasm | Perimeter | 369.31 | 345.54 | 116.18 |
| Cytoplasm | Solidity | 0.64 | 0.64 | 0.08 |
